# Impact of large-scale food fortification programmes on micronutrient inadequacies and their implementation costs: a modelling analysis

**DOI:** 10.1016/S2214-109X(26)00023-9

**Published:** 2026-03-25

**Authors:** Valerie M Friesen, Christopher M Free, Katherine P Adams, Yan Bai, Leah Costlow, Kathryn G Dewey, William Masters, Mduduzi N N Mbuya, Stella Nordhagen, Florencia C Vasta, Ty Beal

**Affiliations:** a Global Alliance for Improved Nutrition, London, UK; b University of California Santa Barbara, Santa Barbara, CA, USA; c University of California Davis, Davis, CA, USA; d The World Bank, Washington, DC, USA; e Tufts University, Boston, MA, USA; f Global Alliance for Improved Nutrition, Washington, DC, USA; g Global Alliance for Improved Nutrition, Geneva, Switzerland

## Abstract

**Background:**

Micronutrient inadequacies are estimated to affect billions of people worldwide, yet these estimates exclude fortification. There are no global estimates of the impact of current fortification programmes on micronutrient inadequacies. We aimed to estimate how improving existing programmes or establishing new ones affects the prevalence of micronutrient inadequacies and to estimate their global costs.

**Methods:**

In this modelling study, we estimated the prevalence of inadequate intakes of 13 micronutrients across 185 countries by integrating global modelled dietary intake data from the Global Dietary Database, with fortification programme parameters from the Global Fortification Data Exchange. We modelled six scenarios: no fortification, current fortification, improved compliance, aligned standards, aligned standards with improved compliance, and aligned standards with improved compliance and expanded coverage. Implementation costs were calculated as the sum of premix, industry-related, and government costs across five fortified foods: wheat flour, maize flour, oil, rice, and salt.

**Findings:**

Compared with no fortification, current fortification programmes prevent 7·0 billion inadequate person-nutrient intakes annually at a global cost of $1·06 billion (mean of $0·18 [SD 0·28] per person, 2021 US$, across the five fortified foods), with iodine fortification alone preventing 3·3 billion inadequacies. Despite current fortification, 38·6 billion inadequate intakes persist. Improving compliance would prevent 13·1 billion inadequacies at a global cost of $3·48 billion annually (mean $0·23 [SD 0·33] per person). Aligning standards and improving compliance would prevent 17·2 billion inadequacies at $6·56 billion annually (mean $0·63 [SD 0·53] per person). Aligning standards, improving compliance, and expanding coverage would prevent 24·7 billion inadequacies at $9·19 billion annually (mean $1·15 [SD 0·58] per person), although 20·9 billion inadequacies would remain.

**Interpretation:**

Fortification is a cost-effective intervention that greatly reduces micronutrient inadequacies, particularly for iodine and iron. Improving compliance offers immediate gains. Aligning with international guidelines and expanding programmes could triple their effects but cannot eliminate all inadequacies, underscoring the importance of complementary approaches to improve diet quality.

**Funding:**

Swiss Agency for Development and Cooperation and the Gates Foundation.

## Introduction

Micronutrient deficiencies are a substantial public health problem and are linked to severe adverse outcomes, such as poor growth, blindness, cognitive impairment, reduced work productivity, and mortality.^1^ Worldwide, about 372 million preschool-aged children (aged 6–59 months) and 1·2 billion non-pregnant women of reproductive age (aged 15–49 years) are deficient in at least one of four key micronutrients (iron, zinc, folate, and vitamin A).^1^ For many micronutrients and population groups, global estimates are unknown due to data scarcity.^1^

In the absence of biomarkers for micronutrient deficiencies, dietary intake data are often used to estimate inadequacies. In 2024, Passarelli and colleagues estimated that more than 5 billion people had inadequate intakes of iodine (68% of the global population), vitamin E (67%), and calcium (66%) and that more than 4 billion people had inadequate intakes of iron (65%), riboflavin (55%), folate (54%), and vitamin C (53%) based on modelled dietary intakes relative to population requirements.^2^ These estimates do not account for the degree of industrial food fortification and could therefore be overestimates for micronutrients that are commonly added to purchased staples, such as iodine in salt.

Large-scale food fortification (LSFF; the addition of micronutrients to commonly consumed staple foods or condiments during processing) is a widely implemented and potentially cost-effective intervention that aims to reduce the prevalence of micronutrient deficiencies.^3^ According to the Global Fortification Data Exchange (GFDx), as of July, 2024, more than 150 countries globally had voluntary or mandatory legislation to fortify at least one staple food or condiment. This legislation includes programmes adding micronutrients to salt (125 mandatory and 21 voluntary), wheat flour (91 mandatory and 13 voluntary), edible oil (34 mandatory and seven voluntary), maize flour (19 mandatory and two voluntary), and rice (eight mandatory and nine voluntary). Extensive evidence shows that well designed and effectively implemented LSFF programmes can make substantial contributions to micronutrient intakes and reduce micronutrient inadequacies and deficiencies while restricting risk of excessive intake.^3^, ^4^, ^5^ Most previous studies have focused on select countries or population groups, such as preschool-aged children and women of reproductive age. As a result, the global impact of fortification among other age and sex groups to date and the potential for increasing that impact remain unknown.

**Research in context**

**Evidence before this study**

A 2024 analysis estimated the global prevalence of inadequate micronutrient intakes using modelled dietary intake data but did not account for the contribution of fortification. The Global Fortification Data Exchange has been compiling global data on fortification standards, compliance, and coverage over the past several years. However, to our knowledge, there have been no global estimates of the impact of fortification on dietary micronutrient adequacy.

**Added value of this study**

This analysis provides, to our knowledge, the first global estimates of inadequate micronutrient intakes that include fortification. We estimate intake inadequacies for 13 micronutrients when including fortification and the associated costs of current fortification programmes and different scenarios with improved fortification standards, compliance, and coverage. This study uses publicly available data and provides all code to make these results accessible to researchers, practitioners, and the public.

**Implications of all the available evidence**

These findings show that improving large-scale food fortification programme compliance with existing standards offers the most immediate benefits for preventing inadequate person–nutrient intakes. In addition, aligning programme standards with international guidelines and expanding coverage could triple their impact. However, even optimal fortification cannot eliminate all inadequacies and carries some risk of excessive intakes, underscoring the importance of overall diet quality for population health. Future research should compare these estimates of inadequacies that include fortification with micronutrient deficiencies to understand how well they reflect biological status.

There is limited, although increasing, evidence of the cost-effectiveness of implementing LSFF programmes. Most of the existing evidence is based on single countries and food vehicles. In 2011, Horton and Mannar provided summary estimates of the cost per consumer in low-income and middle-income countries for multiple food vehicles, but these estimates cover a small set of micronutrients and were not extrapolated up from cost per consumer to total cost.^6^ To our knowledge, there has never been an effort to systematically estimate the global costs of implementing LSFF programmes.

In this study, we build on the approach developed in 2024 for a no-fortification scenario,^2^ estimating the global prevalence of inadequate micronutrient intakes for 13 essential micronutrients across 185 countries (appendix 1 p 12) and accounting for intakes from LSFF programmes as of July, 2024 (ie, current fortification). We aimed to assess the potential effects of improving existing LSFF programmes or establishing new ones on the estimated prevalence of inadequate micronutrient intakes by modelling four improved fortification scenarios (appendix 1 p 13). Finally, we aimed to estimate the global costs of LSFF programmes under current implementation standards and with the four modelled scenarios.

## Methods

### Estimating prevalence of inadequate micronutrient intakes

#### Data sources

**Figure 1:**
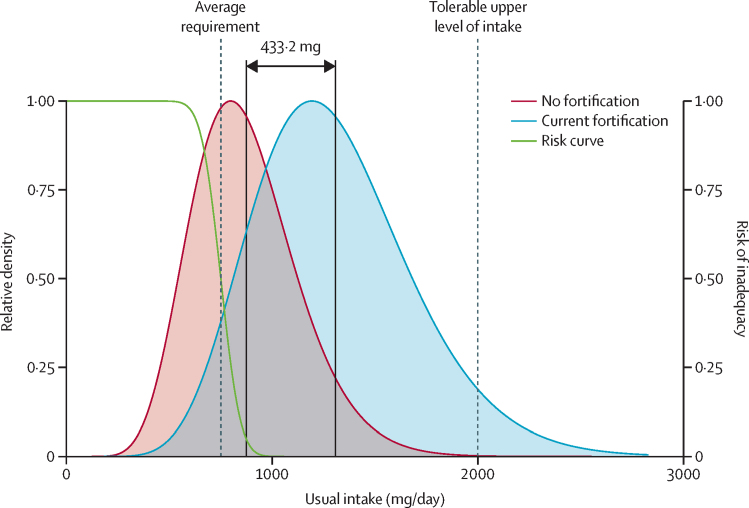
Our approach for estimating the prevalence of inadequate and excess intakes associated with the micronutrient intake distributions resulting from different fortification scenarios This example illustrates the usual calcium intake distribution without fortification (red) and under current fortification (grey) for males aged 60–64 years in Kuwait. We estimate the intake distribution under each fortification scenario by shifting the baseline distribution of the no fortification scenario. The distribution with fortification has the same amount of variability as the distribution without it, but the mean is equal to the mean of the no fortification distribution plus the size of the micronutrient intake contribution resulting from fortification. The solid vertical lines show the mean, and the 433·2 mg indicates the size of the micronutrient intake contribution resulting from fortification. The leftmost vertical dashed line indicates the average requirement and the curved black line indicates the risk of inadequacy associated with the requirement distribution. The intersection of each usual intake distribution and the risk curves is used to estimate the prevalence of inadequate intakes. The rightmost vertical dashed line indicates the tolerable upper level of intake: we estimate the prevalence of excess intakes as the proportion of the population with intakes above this level. Current fortification programmes are those operational at the time of writing. Relative density is the density of each intake distribution divided by its maximum value, which scales both distributions between 0 and 1.

We used the subnational micronutrient intake distributions estimated by Passarelli and colleagues in 2024 as our baseline intake distributions.^2^ These intake distributions cover 15 micronutrients across 34 age–sex groups in 185 countries, which account for 99·3% of the global population. The medians of the distributions are derived from the Global Dietary Database and the shapes of the distributions are derived from dietary recall surveys (the data are from 1990 to 2018, but the modelled estimates are for 2018).^7^ These skewed distributions follow log-normal or gamma distributions, which enable the estimation of inadequate and excess intakes using the probability approach (figure 1).^8^

**Figure 2:**
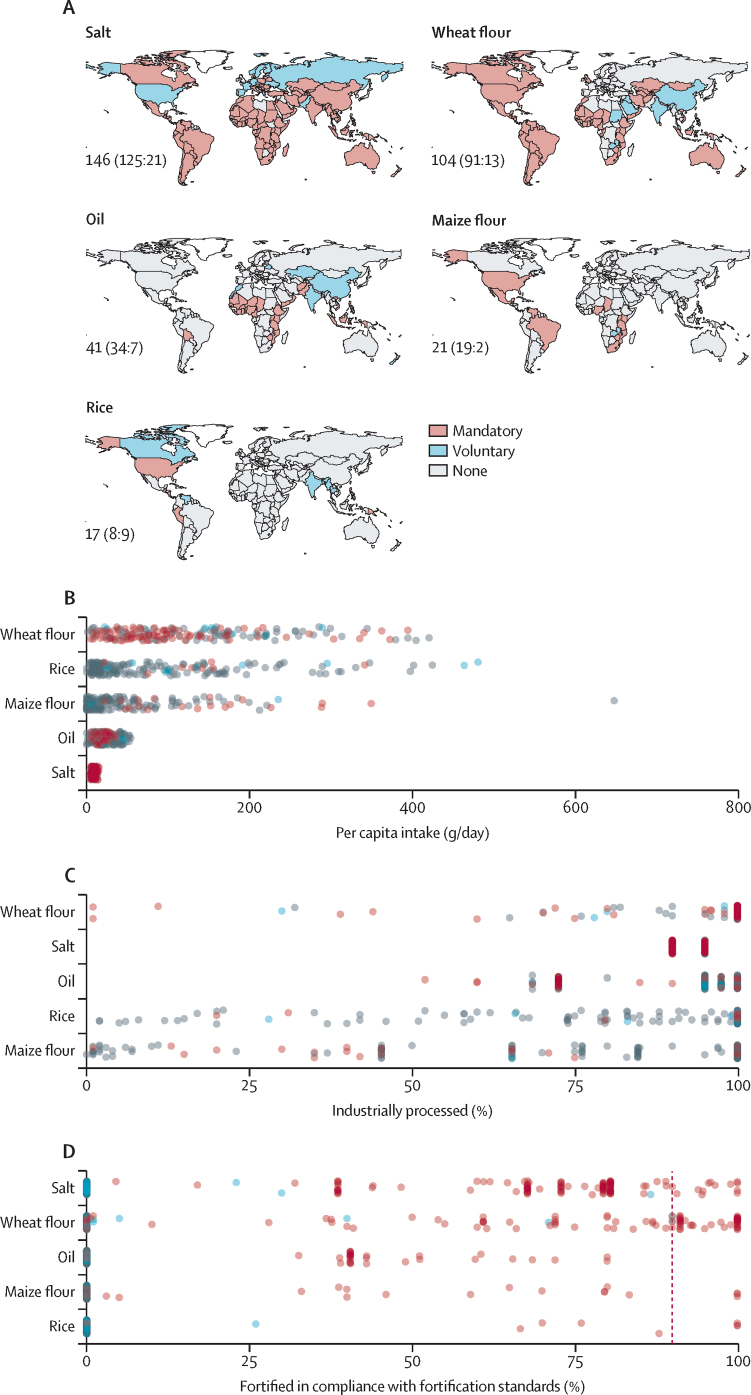
Characteristics of micronutrient fortification programmes for five food vehicles (A) Current country fortification programme status (mandatory, voluntary, or none). The number of countries with mandatory or voluntary fortification programmes is reported as total (mandatory:voluntary). Daily per capita intake (B), level of industrial processing (C), and compliance with existing fortification standards (when applicable; D) are shown for each food vehicle. The vertical red line in graph D marks the 90% compliance level assumed in the improved compliance fortification scenarios. Current fortification programmes are those operational at the time of writing.

We used data from the GFDx, which provides coverage and compliance data for mandatory and voluntary fortification programmes, to estimate micronutrient intake contributions from current and improved fortification programmes (figure 2). Specifically, we extracted the following indicators from the GFDx for five commonly fortified food vehicles (wheat flour, maize flour, oil, rice, and salt): (1) fortification programme status (mandatory or voluntary), (2) daily per capita intake, (3) percentage of food vehicles that are industrially processed, (4) percentage of industrially processed food vehicles that are in compliance with fortification standards, and (5) micronutrient levels and compounds in the current fortification standards (figure 2; appendix 1 pp 25–31). We excluded fluoride (since fluoridation is rare) and vitamin D (the requirements for which can be partly met through sun exposure; appendix 1 p 12). We imputed missing values for the percentage of food that is industrially processed (100% of salt values, 85% of oil, 29% of maize flour, 24% of rice, and 4% of wheat flour) and the percentage that is fortified in compliance with fortification standards (salt 73%, oil 68%, rice 29%, wheat flour 27%, and maize flour 19%) using procedures described in appendix 1 (pp 32–34).

#### Fortification scenarios

We evaluated six fortification scenarios (appendix 1 p 13). The no fortification scenario is based on estimates of inadequate intakes from Passarelli and colleagues (2024),^2^ which excluded fortification. This scenario acts as a baseline against which we can estimate the benefits of current and improved fortification programmes. The current fortification scenario is based on current levels of compliance with mandatory and voluntary fortification standards as documented by the GFDx. The four improved fortification scenarios were: improving industry compliance while retaining existing standards (ie, improved compliance); aligning existing standards to reflect international fortification guidelines with no changes to industry compliance (ie, aligned standards); aligning existing standards to reflect international fortification guidelines while improving industry compliance (ie, aligned and improved); and establishing aligned standards across countries that reflect international fortification guidelines—targeting micronutrients with high country-level baseline inadequacies and appropriate food vehicles—while also improving industry compliance (ie, aligned, improved, and expanded). The improved compliance scenario considers the benefits of improved compliance at 90% for all fortification programmes (both mandatory and voluntary; it is currently less than 90% [figure 2]). Two of the scenarios consider the benefits of aligning fortification standards with international fortification guidelines^9^, ^10^, ^11^ in terms of micronutrient levels and compounds (appendix 1 pp 15–16, 35–36). These benefits are evaluated under both current (ie, aligned standards) and improved compliance (ie, aligned standards and improved compliance) scenarios to estimate the marginal benefits provided. The final scenario of aligned standards, improved compliance, and expanded coverage assesses benefits from expanding aligned fortification standards with 90% compliance to targeted food vehicles that deliver micronutrients in countries with high prevalence of inadequate intakes in the no fortification scenario (appendix 1 pp 3–4).

#### Estimation approach

For each scenario, we calculated the micronutrient contribution from fortification as:

$${\text{I}}_{msca}=\sum_{\text{FV}}^{5}F_{csa}\times {\text{IP}}_{\text{FV}c}\times {\text{FC}}_{\text{FV}c}\times D_{\text{FV}cm}$$

where *I*_mcsa_ is the daily intake contribution of micronutrient (*m*) for sex (*s*) and age group (*a*) in a country (*c*) resulting from all five food vehicles (FV). The micronutrient intake contribution of each food vehicle is the product of its daily per capita intake (*F*_csa_), the proportion of the food vehicle that is industrially processed (IP_FVc_), the proportion of the food vehicle that is processed in compliance with fortification standards (FC_FVc_), and the amount of the micronutrient added through fortification (*D*_FVcm_). Age–sex specific daily intakes (*F*_csa_) were estimated by scaling GFDx's population-level estimates using age–sex caloric intake estimates from Springmann^12^ (appendix 1 p 37).

We estimated the shift in the prevalence of inadequate and excess intakes resulting from each fortification scenario by the following three methods: adding the fortification contribution to the mean of the unfortified intake distribution; shifting the unfortified distribution to the new mean while retaining the original shape; and using the probability approach^8^ to estimate the prevalence of inadequate and excess intakes (figure 1). We assessed intake inadequacy using the harmonised average requirements from Allen and colleagues (2020)^13^ for most micronutrients (appendix 1 pp 38–39). To assess the risk that food fortification causes adverse health effects from excess micronutrient intakes, we estimated the percentage of each subpopulation exceeding the tolerable upper level of intake (UL; figure 1) for the seven micronutrients with harmonised ULs used by Allen and colleagues^13^ (appendix 1 pp 17, 38–39). This was done using the *shift_dist(), sev()*, and *above_ul()* functions in the *nutriR* package. We calculated the number of people with inadequate or excess intakes using estimates of population size for each country, sex, and age group from the World Bank.^14^ These methods are deterministic and do not estimate uncertainty resulting from, among other factors, the imputation of missing information on fortification programmes and intake distributions.

### Estimating costs of implementing the fortification scenarios

Detailed methods and data sources used to estimate LSFF programme costs are available in appendix 1 (pp 6–9). We estimated the total annual cost of each LSFF programme under each scenario as the sum of premix, industry-related, and government costs. We estimated premix costs per metric tonne of food vehicle using a premix cost calculator populated with data on the micronutrient level, micronutrient compound, and activity level (ie, the proportion of micronutrient in the micronutrient compound). Global micronutrient compound prices and activity levels were informed by consultations in 2023 and 2024 with several industry experts with experience across diverse contexts in premix procurement, supply, and prices. We adjusted estimates of the cost of premix per tonne for international shipping, taxes and duties, and domestic transport, storage, and handling. To estimate total premix costs, we multiplied the premix cost per tonne by the estimated annual quantity of fortified food vehicle in the food system. Using a rough estimate of the number of domestic industrial processing facilities for each food vehicle in each country, we estimated annual industry-related fortification costs as the sum of annualised fortification and quality assurance–quality control (QA–QC) equipment costs, QA–QC supply costs, labour costs for fortification and QA–QC, training costs, and management, overhead, and administration costs. For existing LSFF programmes, we estimated annual government-related costs as the sum of annualised monitoring equipment costs; monitoring supply costs; labour costs for industry, import, and commercial monitoring; social marketing costs; training costs; and management, overhead, and administration costs. For new LSFF programmes, government-related costs also included annualised planning and launching costs.

### Sensitivity analyses

We analysed the sensitivity of our results for inadequate intakes and implementation costs to the alignment of calcium fortification standards with international standards for wheat flour, because calcium fortification was found to be a major driver of cost but is seldom done. We also analysed the sensitivity of these results to different estimates of fortification compliance that were based on highly uncertain proxy estimates. Finally, we analysed the sensitivity of implementation costs to premix costs (appendix 1 pp 9–10).

### Role of the funding source

The funders of the study had no role in the study design, data collection, data analysis, data interpretation, or writing of the report.

## Results

**Figure 3:**
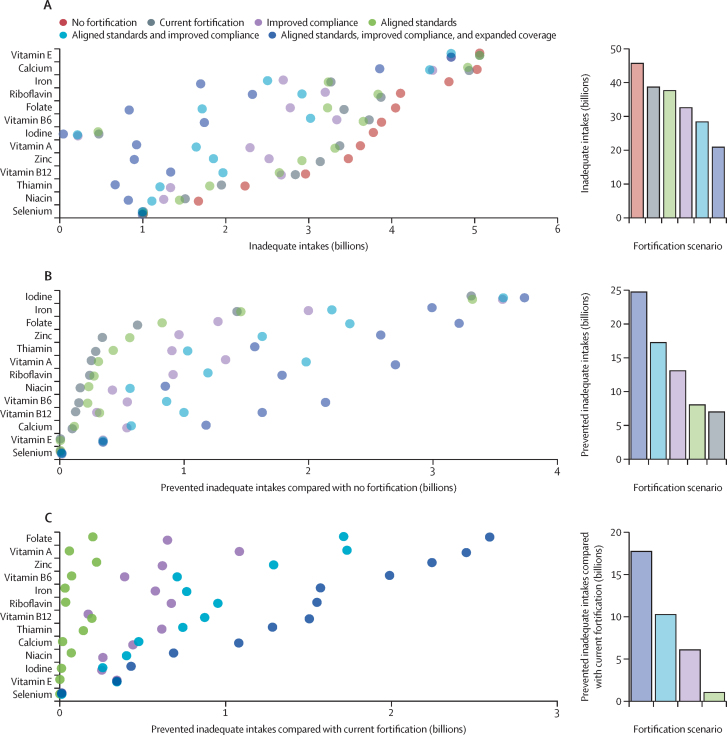
Effects of current and improved fortification programmes on the global prevalence of inadequate intakes In the scatter graphs, points are slightly transparent and vertically staggered to expose overlapping points. The bar graphs show the sum of the metrics across all evaluated nutrients for each fortification scenario. (A) The number of inadequate person–nutrient intakes under each fortification scenario; nutrients are sorted by the prevalence of inadequate intakes under the no fortification scenario. (B) The number of inadequate person–nutrient intakes prevented by each fortification scenario compared with the no fortification scenario; nutrients are sorted by the prevalence of inadequate intakes under the current fortification scenario. (C) The number of inadequate person–nutrient intakes prevented under each improved fortification scenario compared with current fortification; nutrients are sorted by the prevalence of inadequate intakes under the aligned standards, improved compliance, and expanded coverage scenario. Current fortification programmes are those operational at the time of writing.

**Figure 4:**
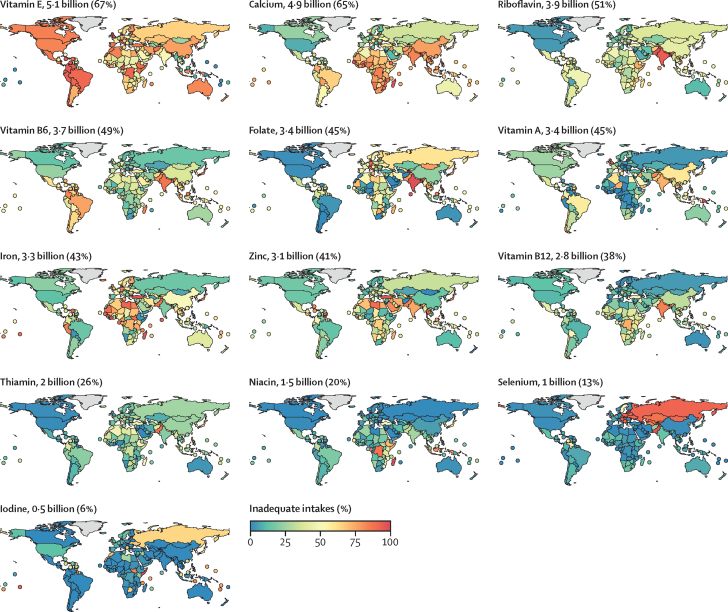
Estimated prevalence of intake inadequacies, accounting for current fortification by country and micronutrient The estimated number and proportion of the global population with inadequacies is labelled above each map. Micronutrients are sorted in order of decreasing inadequate intakes. Countries with land areas less than 25 000 km^2^ are shown as points to increase visibility.

We estimate that, as of 2024, fortification programmes prevent 7·0 billion inadequate person–nutrient intakes (ie, the number of people with inadequate intake of each of the 13 individual micronutrients summed together; hereafter referred to as inadequate intakes; Figure 3, Figure 4; appendix 1 pp 18, 40). Current iodine fortification programmes are especially effective and are estimated to eliminate 3·3 billion inadequate intakes (figure 3B) in the 139 countries with iodine fortification programmes (figure 2A) and reduce the estimated global population with inadequate iodine intake from 3·8 billion to 473·0 million people (figure 3A). Current iron fortification programmes are also highly effective because they are estimated to prevent inadequate iron intakes for 1·4 billion people. For other micronutrients, we estimate that current fortification programmes prevent inadequate intakes for folate (621 million people), zinc (338 million), thiamin (284 million), vitamin A (249 million), riboflavin (236 million), niacin (160 million), vitamin B6 (149 million), vitamin B12 (123 million), calcium (96 million), vitamin E (310 000), and selenium (283 000). The benefits of current fortification vary by country (appendix 1 p 40) based on dietary intakes of micronutrients and the parameters of current fortification programmes (figure 2). Despite the programmes' strengths, they do not eliminate all inadequacies: after accounting for current fortification programmes, an estimated 38·6 billion inadequate intakes persist (Figure 3, Figure 4; appendix 1 p 18).

Improving compliance with current fortification programmes to at least 90% would prevent an additional 6·1 billion inadequate intakes (or 13·1 billion inadequacies versus no fortification; Figure 3, Figure 4; appendix 1 pp 18–40). The largest gains would come through vitamin A fortification, for which increased compliance would prevent an additional 1·1 billion inadequate intakes. Increased compliance with current fortification programmes would prevent additional inadequate intakes for riboflavin (672 million), folate (648 million), zinc (618 million), thiamin (672 million), iron (575 million), calcium (440 million), vitamin B6 (390 million), vitamin E (343 million), niacin (259 million), iodine (253 million), vitamin B12 (171 million), and selenium (11 million; figure 3C; appendix 1 p 18). The benefits of improved compliance with current fortification programmes vary by country (figure 2; appendix 1 p 40). Despite improved compliance with current fortification programmes, an estimated 32·5 billion inadequate intakes would remain (figure 3A; appendix 1 p 18).

Aligning fortification standards with international fortification guidelines while maintaining current programme compliance would only prevent an additional 1·0 billion inadequate intakes compared with current fortification (Figure 3, Figure 4; appendix 1 pp 18, 40), which is much less than the 6·1 billion prevented through improved compliance. This low estimate is because current fortification standards are already higher than international guidelines in many countries (appendix 1 pp 35–36).

Both aligning fortification standards and improving compliance to at least 90% would prevent 17·2 billion inadequacies compared with no fortification, an additional 10·3 billion inadequate intakes compared with current fortification, and an additional 4·2 billion inadequate intakes compared with improved compliance with current fortification standards (Figure 3, Figure 4; appendix 1 p 18, 40). Aligned fortification standards with improved compliance would prevent additional inadequate intakes compared with current fortification for: vitamin A (1·73 billion), folate (1·71 billion), zinc (1·3 billion), thiamin (741 million), riboflavin (952 million), vitamin B12 (873 million), iron (764 million), vitamin B6 (707 million), calcium (475 million), niacin (401 million), vitamin E (343 million), iodine (258 million), and selenium (11 million; figure 3C; appendix 1 p 18). The benefits of aligned fortification standards with improved compliance vary by country (figure 2; appendix 1 p 40). Despite the adoption of aligned fortification standards with improved compliance, an estimated 28·4 billion inadequate intakes would remain (figure 3A; appendix 1 p 18).

In addition to aligning fortification standards and improving compliance to at least 90%, we estimated that expanding the number of fortification programmes implemented to include new programmes in countries where there is an identified need and appropriate food vehicle would prevent up to 24·7 billion inadequate intakes: an additional 17·7 billion inadequate intakes prevented compared with current fortification and an additional 7·5 billion inadequate intakes prevented compared with aligned fortification standards with improved compliance (Figure 3, Figure 4; appendix 1 pp 18, 40). Aligned standards, improved compliance, and expanded fortification programmes would prevent additional inadequate intakes compared with current fortification for folate (2·6 billion), vitamin A (2·5 billion), zinc (2·2 billion), vitamin B6 (2·0 million), iron (1·6 billion), riboflavin (1·5 billion), vitamin B12 (1·5 billion), thiamin (1·3 billion), calcium (1·1 billion), niacin (685 million), iodine (429 million), vitamin E (343 million), and selenium (11 million; figure 3C; appendix 1 p 18). The benefits of aligned standards, improved compliance, and expanded fortification programmes vary by country (figure 2; appendix 1 p 40). However, even after aligning standards, improving compliance, and expanding fortification programmes, an estimated 20·9 billion inadequate intakes would remain (figure 3A; appendix 1 p 18).

**Figure 5:**
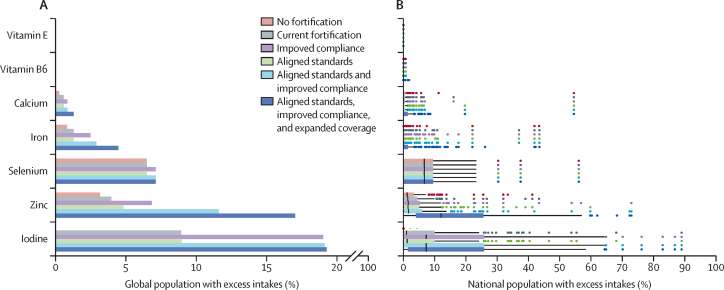
Populations at risk of adverse effects from usual micronutrient intakes above the tolerable upper level of intake (A) Percentage of global population. (B) Percentage of national populations. Micronutrients are sorted by the average percent of the population above the upper level of intake across all five scenarios. In the boxplots (B), the solid line indicates the median across all 185 countries, the box indicates the IQR, the whiskers indicate 1·5 times the IQR, and points indicate outliers. Current fortification programmes are those operational as of July, 2024.

The prevalence of excess intakes under the different scenarios was estimated to vary widely by micronutrient. For example, it was estimated to be rare for vitamin E and B6 and uncommon for calcium (figure 5) because the ULs for these micronutrients greatly exceed usual intakes (appendix 1 p 38). The prevalence of excess iodine intakes is negligible without fortification but, with high iodine fortification, increases significantly under each fortification scenario (figure 5). Selenium and zinc have the next highest prevalences of excess intakes after iodine, whereas vitamins E and B6 have the lowest prevalences of excess intakes, followed by calcium and iron (figure 5). The results vary by country, with some countries showing very high prevalences of excess intakes (figure 5).

**Figure 6:**
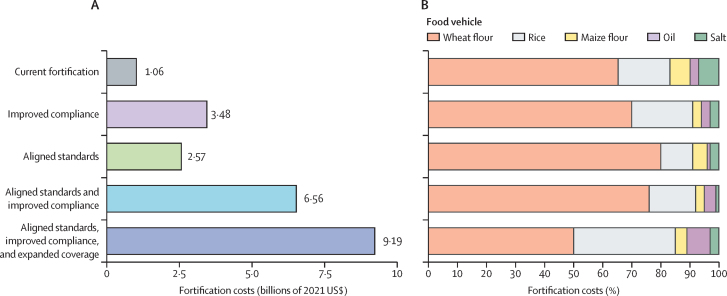
Global annual costs of fortification (A) Global annual costs of fortification. (B) Percent share of global annual costs by food vehicle. Cost estimates include premix, industry-related, and government-related costs for wheat flour, rice, maize flour, oil, and salt fortification. Food vehicles are sorted by their average share of costs across fortification scenarios. Current fortification programmes are those operational as of July, 2024.

The estimated global annual cost of current fortification programmes is $1·06 billion (2021 USD; figure 6). This cost translates to an annual cost per person of just $0·12 for wheat flour, $0·06 for maize flour, $0·10 for rice, $0·02 for refined oil, and $0·01 for salt, which equates to a mean cost of $0·18 (SD 0·28) per person across the five fortified foods (appendix 1 p 19). If these programmes reached 90% compliance, the annual cost would rise to $3·48 billion or a mean cost of $0·23 (0·33) per person annually. If standards were aligned with international guidelines, the estimated annual cost of current fortification programmes would be $2·57 billion for current compliance (at a mean cost of $0·52 [0·50] annually), and $6·56 billion for improved compliance (mean $0·63 [0·53]). The estimated global annual cost of the aligned, improved, and expanded scenario is $9·19 billion, or a mean of $1·15 (0·58) per person annually. In each scenario, between 66% and 80% of the global annual cost of fortification is attributable to the cost of wheat flour fortification (figure 6). Rice fortification contributes to 11–22% of the total annual cost and maize flour, refined oil, and salt each contribute to 1–8% of the total.

The results of the sensitivity analyses indicate marginal effects on global estimates of inadequate intakes (appendix 1 pp 41–42). However, costs of fortification programmes are proportionally sensitive to estimates of compound prices (appendix 1 p 43) and are much higher when calcium is aligned with wheat flour standards (appendix 1 p 44). Across all scenarios and all food vehicles other than salt, micronutrient premix represents at least 70% of the total cost. The estimated global cost of fortification changes substantially (by 44–48%) if the price of all micronutrient compounds is varied down or up by 50% (appendix 1 p 10).

## Discussion

To the best of our knowledge, our analysis provides the first global estimates of the impacts and costs of LSFF programmes. We estimate that existing LSFF programmes for staple foods and salt prevent approximately 7·0 billion inadequate micronutrient intakes annually at an annual average total cost of $0·18 per person, with iodine and iron fortification accounting for two-thirds of these benefits. However, substantial gaps remain: an estimated 38·6 billion inadequate intakes persist under current programmes due to poor quality diets and suboptimal fortification guidelines, compliance, and programme coverage. By improving compliance with existing standards, aligning fortification guidelines, and expanding programmes to countries with high inadequacy prevalence and suitable food vehicles, LSFF programmes could prevent up to 24·7 billion inadequate intakes at an average cost of $1·15 per person annually.

Our findings build on previous estimates of global micronutrient inadequacies without accounting for fortification.^2^ By integrating LSFF contributions, we reveal that current programmes reduce iodine inadequacy by 87% compared with no iodine fortification, aligning with global reports of substantial reduction of iodine deficiency disorders in countries with sustained salt iodisation.^15^ Similarly, iron fortification prevents 1·4 billion inadequacies, which is consistent with meta-analyses showing LSFF's efficacy in reducing iron deficiency and anaemia.^16^

Current LSFF programmes cost $1·06 billion annually—just $0·18 per person on average—with salt iodisation being the most economical ($0·01 per person). These figures align with Horton and Mannar's 2011 estimates^6^ but extend them globally, showing scalability. Even under the most resource-intensive scenario ($9·19 billion annually or $1·15 per person on average), costs remain modest compared with the tens of billions estimated in annual economic losses attributed to micronutrient deficiencies.^17^

Although LSFF programmes substantially reduce micronutrient inadequacies, our analysis highlights context-specific risks of excessive intakes, particularly for iodine, zinc, selenium, and iron. Improving compliance, aligning guidelines, and expanding programmes could place more than 15% of the global population at risk of excess intakes of iodine (which can lead to thyroid dysfunction) and zinc (which can interfere with copper absorption). Risk of excessive intakes due to LSFF programmes is low for other micronutrients. These findings emphasise the importance of ensuring that national fortification standards are set based on population micronutrient needs and baseline dietary intakes and have monitoring systems to balance the benefits and risks—a principle underscored in WHO guidelines but inconsistently implemented.^18^

Even after improving compliance and standards, an estimated 20·9 billion inadequate intakes would remain, highlighting the importance of working towards healthy diets for all to sustainably eliminate micronutrient deficiencies. High-quality diets that include a diverse range of nutrient-dense foods can meet most micronutrient needs and provide additional beneficial compounds bound within a complex food matrix. Yet such diets remain unaffordable for 2·6 billion people worldwide.^19^ Furthermore, pregnant and lactating people and children aged 6–23 months might require additional interventions to meet their increased micronutrient needs during these crucial life stages, such as supplementation or targeted fortified foods.

Three interconnected policy actions could amplify LSFF's benefits while mitigating risks. First, improving compliance with existing standards must be prioritised. Raising industry compliance with current programmes to 90% could prevent 6·1 billion additional inadequacies for an average cost of $0·23 per person annually. Achieving this compliance requires addressing technical barriers, such as inconsistent premix supply chains and equipment malfunctions, through public–private partnerships and investments in QA–QC. Second, aligning national fortification standards with WHO guidelines would address underfortification of key micronutrients. Many countries currently fortify wheat flour with iron at levels less than WHO recommendations, thereby limiting their effects. Adopting harmonised standards in addition to improved compliance could prevent an additional 4·2 billion additional inadequacies at an average cost of $0·63 per person annually. Third, for the greatest impact, in addition to improving compliance and aligning standards, expanding programmes to high-need regions with suitable food vehicles is essential. Expanding programmes could prevent an additional 7·5 billion inadequacies compared with aligned fortification standards and improved compliance, at an average cost of $1·15 per person annually. For instance, scaling rice fortification in south Asia—where the majority of the population consumes rice daily—could address folate inadequacies affecting hundres of millions of people. New programmes should prioritise countries with widely consumed, industrially processed, staple foods to ensure cost-efficient delivery.

Our study has several limitations. First, the Global Dietary Database uses fixed calorie intakes across countries, which might misestimate nutrient intakes. Second, reliance on the Food and Agricultural Organization of the UN's national food balance sheets in the GFDx could misestimate intake of fortified foods because they do not account for small-scale production or food waste.^2^ Third, compliance estimates from proxy sources (eg, expert opinion) might inflate impact projections, although sensitivity analyses showed marginal effects on global results. Fourth, we excluded biofortification and supplementation programmes, such as multiple micronutrient supplementation; future programmes and policies should integrate these interventions to improve access to different forms of micronutrients and to minimise risks of excessive intakes. Fifth, we were unable to propagate uncertainty or produce estimates of variation because the data required to quantify key sources of uncertainty—such as variability in fortification coverage, intake distributions, and compliance—are largely unavailable or incomplete across countries, highlighting an essential gap for future research. Sixth, for 1·3% of the global population, age-specific and sex-specific calorie intake estimates were unavailable and we substituted values from other countries. Although these substitutions could introduce bias—particularly for countries with systematically different diets or living standards—they make up a very small share of the global population and are unlikely to substantially affect our global estimates. Finally, our analysis uses static premix prices, despite potential scale economies that could reduce costs by up to 48%, as shown in sensitivity analyses.

LSFF is a cornerstone of global nutrition strategies, offering a cost-effective, medium-term solution to micronutrient deficiencies and their associated adverse health outcomes at a time when healthy diets with diverse foods remain out of reach for billions of people. Current programmes already avert many micronutrient inadequacies but their potential is far from exhausted. By improving compliance, aligning standards, and expanding coverage, policy makers can bridge existing gaps, reduce unintended health consequences, and accelerate progress towards Sustainable Development Goal 2, which aims to eliminate hunger by 2030. Future research could further strengthen the evidence base by consolidating global data on supplementation and biofortification programmes. In addition, countries in which the modelling shows substantial potential for reductions in inadequacies present opportunities for deeper analyses using richer dietary data and targeted primary data collection to improve estimates of coverage and cost, as well as the overall impact on the global health burden (eg, in terms of years of life lost to illness, disability, or early death). Long-term success, however, hinges on parallel investments in dietary diversity, supplementation, and robust monitoring systems to ensure equitable, safe access to essential micronutrients.

### Contributors

### Equitable Partnership Declaration

### Data sharing

All data and code are available on GitHub and Dataverse.

## Declaration of interests

We declare no competing interests.

## Supplementary Materials

Supplementary appendix 1

Equitable Partnership Declaration
