# Supplementary material for: Impact of large-scale food fortification programmes on micronutrient inadequacies and their implementation costs: a modelling analysis
Source: Lancet Glob Health. 2026 Mar 25;14(5):e762–71. doi: 10.1016/S2214-109X(26)00023-9 (PMC13106045; doi:10.1016/S2214-109X(26)00023-9)
Supplement: Supplementary appendix 1 [file mmc1.pdf]

THE LANCET
Global Health

Supplementary appendix 1

This appendix formed part of the original submission and has been peer reviewed. We post it as supplied by the authors.

Supplement to: Friesen VM, Free CM, Adams KP, et al. Impact of large-scale food fortification programmes on micronutrient inadequacies and their implementation costs: a modelling analysis. *Lancet Glob Health* 2026; published online March 25. https://doi.org/10.1016/S2214-109X(26)00023-9.

Table of Contents

Table of Contents 1
Supplemental Methods 2
Detailed methods for estimating prevalence of intake inadequacy 2
Data 2
Fortification scenarios 3
Modelling approach 4
Detailed methods and data sources for estimating costs of implementing the fortification scenarios 6
Micronutrient premix costs 6
Industry-related costs 7
Government-related costs 8
Sensitivity analyses 9
Supplemental Results 10
Cost analysis 10
Sensitivity analyses 10
Compound prices 10
Aligned standard for calcium in wheat flour 10
Use of proxy values for fortification compliance 11
Supplemental Tables 12
Supplemental Figures 25
References 46

Supplemental Methods

Detailed methods for estimating prevalence of intake inadequacy

Data

We used the subnational micronutrient intake distributions estimated by Passarelli et al. (2024)[1] as the baseline intake distributions for our analysis. Passarelli et al. (2024)[1] parameterized skewed usual intake distributions for 15 micronutrients in 34 age-sex groups (2 sexes x 16 5-year age groups and an 80+ age group) in 185 countries, representing 99.3% of the global population. The median of these distributions is equivalent to that estimated by the Global Dietary Database, and the shape of these distributions was informed by dietary recall surveys from 31 countries.[2] Importantly, the Global Dietary Database does not account for fortification in its estimates of micronutrient intakes, which means that the Passarelli et al. (2024)[1] intake distributions and associated estimates of inadequate intakes do not account for fortification. The usual intake distributions parameterized by Passarelli et al. (2024)[1] follow either a log-normal or gamma probability distribution, which allows for the estimation of inadequate micronutrient intakes through the probability approach.[3] Passarelli et al. (2024)[1] also developed an algorithm for shifting the median of an intake distribution while retaining its shape as an approach to simulating the impacts of a public health intervention such as fortification (**Fig. 1**). This functionality is implemented through the *nutriR* package,[4] which we use to shift the baseline distributions based on the micronutrient contributions resulting from fortification and to estimate the change in micronutrient intake inadequacies resulting from this shift.

We used data from the GFDx to estimate the micronutrient intake contributions resulting from current and improved large-scale food fortification programs (**Fig. 2**). Specifically, from the GFDx, we obtained information on the following key traits of all current fortification programs for five commonly fortified food vehicles (i.e., wheat flour, maize flour, oil, rice, and salt): (1) the presence or absence of a mandatory or voluntary food fortification program (GFDx Indicators 1 and 9), (2) the food intake/availability (i.e., the average amount of food that is available for human consumption per capita per day), based on the most recent Food and Agriculture Organization (FAO) food supply data[5] for wheat flour, maize flour, oil, and rice and other sources[6,7] for salt (GFDx Indicator 10), (3) the percentage of food that is industrially processed (GFDx Indicator 12), (4) the percentage of food that is fortified in compliance with fortification standards (based on data from industry compliance assessments or proxies of fortification quality from market/household assessments or expert opinion; GFDx Indicator 15), and (5) the micronutrient levels and compounds in the current fortification standard (i.e., the required amounts and allowable compounds of micronutrient at the point of production/import for the food; GFDx Indicators 6 and 7) (**Figs. 2 & S1-S6**). All data were accessed on 30 July 2024. Although the GFDx data documented 15 micronutrients in the fortification standards across the five fortified foods (**Table S1; Figs. S3-S6**), we only included 13 micronutrients in this analysis. We excluded fluoride (which is included in fortification standards for salt in 21

countries) because its intake distributions were not modelled by Passarelli et al. (2024)[1], and we excluded vitamin D (which is included in fortification standards for oil, wheat flour, rice, and maize flour in 13, 8, 3, and 1 countries, respectively) because its requirements can be partially met through sun exposure[8], which is challenging to account for in global analyses.

We used the GFDx value for the most recent year with data for each key trait (**Fig. S7**). Because the GFDx contained some missing values for the percentage of food that is industrially processed and the percentage of food that is fortified in compliance with fortification standards (**Fig. S8-S9**), we developed procedures for imputing missing values. Data for the percentage of food that is industrially processed were missing for varying percentages across foods: salt (missing for 100% of countries), oil (85%), maize flour (29%), rice (24%), and wheat flour (4%) (**Fig. S8**). We therefore assumed that 95% of salt is industrially processed in high-income and upper-middle-income countries and that 90% of salt is industrially processed in low-income countries (**Fig. S10C**). For the remaining foods, we used the average value for, in order of preference, the World Bank region or continent (**Fig. S10AB**). Data for the percentage of food that is fortified in compliance with fortification standards were similarly missing across foods: salt (missing for 73% of countries), oil (68%), rice (29%), wheat flour (27%), and maize flour (19%) (**Fig. S9**). For voluntary fortification programs missing this information, we assumed 0% compliance. For mandatory fortification programs missing this information, we used the average value for, in order of preference, the World Bank region, continent, or income category (**Fig. S10**). This approach is conceptually similar to that used by Wessells et al.[9] to impute missing compliance estimates for zinc fortification programs.

Fortification scenarios

We evaluated six fortification scenarios (**Table S2**): (a) a "no fortification" scenario, which acts as a baseline for estimating the benefits of current and improved fortification programs; (b) a "current fortification" scenario, which seeks to document the benefits of current fortification programs; and (c) four "improved fortification" scenarios, which seek to reveal the benefits of potential strategies for improving fortification programs by increasing compliance and/or by increasing existing micronutrient levels and/or including additional micronutrients in fortification standards. The "improved compliance" scenario, the first of the improved fortification scenarios, considers the benefits of improving fortification compliance with existing fortification standards in terms of micronutrient levels. The scenario maintains current fortification standards but elevates compliance to 90% for the 258 programs (78.4% of total) with compliance lower than 90% (**Figure 2D**).

The "aligned standards" scenarios, the second and third category of the improved fortification scenarios, seek to understand the benefits of aligning existing country fortification standards with international fortification guidelines in terms of micronutrient levels and compounds (**Table S3 & S4; Figs. S11 & S12**). We evaluated these aligned standards under both current (the "aligned

standards" scenario) and improved compliance (the "aligned and improved" scenario) so that we could measure the marginal benefits resulting from aligning standards alone or aligning standards and improving compliance to 90%. The aligned standards were set based on the recommended micronutrient levels and compounds in international guidelines, i.e., the World Health Organization (WHO) guidelines for fortification of wheat flour[10], maize flour[11], and salt[12]. For wheat flour and maize flour, we selected NaFeEDTA as the chemical form of the compound (assuming a fixed bioavailability of 10% independent of the diet) and assumed low flour extraction rates for iron and zinc. Because the WHO guideline for fortification of rice[13] does not provide specific recommendations on which micronutrients to include and there is no international guideline for fortification of oil, we defined aligned standards for those two foods based on the median of the current country fortification standards for micronutrients that are fortified in at least 5 countries. Furthermore, in some countries, the current fortification standard included additional micronutrients or micronutrients at levels higher than what was specified in the aligned standards as defined above (**Figs. S11 & S12**). In those cases, we retained the current fortification standard in the aligned standards scenarios for that country.

The "aligned, improved, and expanded" scenario, the final improved fortification scenario, seeks to understand the benefits of implementing fortification programs using aligned standards and improved compliance (90%) in countries where there is an identified need (based on the "no fortification baseline" scenario) and an appropriate food vehicle (based on food intake/availability and proportion of food that is industrially processed) in addition to the "aligned and improved" scenario. Specifically, after implementing the "aligned and improved" scenario described above, for countries with ≥ 20% country-level prevalence of inadequate intakes for iodine before fortification, we implemented aligned fortification standards at 90% compliance for salt if the country-level food intake/availability was ≥ 3 g/capita/day and the proportion of food that is industrially processed was ≥ 50%. For other micronutrients with ≥ 20% country-level prevalence of inadequate intake before fortification, we implemented aligned fortification standards at 90% compliance for all foods that deliver the specific micronutrients if the country-level food intake/availability was ≥ 50 g/capita/day for wheat flour, ≥ 50 g/capita/day for maize flour, ≥50 g/capita/day for rice, and/or ≥ 10 g/capita/day for oil and the proportion of food that is industrially processed for each food was ≥ 50%.

Modelling approach

Across all the food fortification scenarios, we calculated the micronutrient contribution from fortification for each subnational age-sex group using the following formula:

$$I_{m,c,s,a} = \sum_{FV}^{5} F_{c,s,a} * IP_{FV,c} * FC_{FV,c} * D_{FV,c,m}$$

Where $I_{m,c,s,a}$ is the micronutrient intake contribution of micronutrient m for sex s and age group a in country c resulting from consumption of the five fortified food vehicles (FV). The micronutrient intake contribution from each food vehicle is calculated by multiplying the per capita intake of the food vehicle ($F_{c,s,a}$), the proportion of the food vehicle that is industrially processed ($IP_{FV,c}$), the proportion of the food that is fortified in compliance with fortification standards ($FC_{FV,c}$), and the amount of the micronutrient (g/kg) added through fortification ($D_{FV,c,m}$; i.e., the micronutrient level in the fortification standard).

The GFDx reports the average daily per capita intake/availability of each food vehicle aggregated across all age-sex groups. To estimate the daily per capita intake/availability of each food vehicle for individual age-sex groups ($F_{c,s,a}$), we scaled the aggregate value by the relative caloric intake of each age-sex group using estimates from Springmann.[14] Springmann[14] estimated caloric intakes of 10 sex-age groups (2 sexes x 5 age groups) for most countries (**Fig. S13**). For the 17 countries without caloric intake data (**Table S8**), we borrowed estimates from a neighboring country in the same region (**Fig. S10**). We then calculated the daily per capita intake/availability of each food vehicle for each individual age-sex group as follows:

$$F_{c,s,a} = F_c \times \frac{K_{c,s,a}}{mean(K_c)}$$

Where F_c is the average aggregated daily per capita intake/availability of a food vehicle reported in the GFDx and $K_{c,s,a}$ is the caloric intake of sex s and age group a in country c from Springmann[14].

We calculated the shift in the prevalence of inadequate micronutrient intakes resulting from each fortification scenario by: (1) adding the micronutrient contribution resulting from fortification to the mean of the unfortified micronutrient intake distribution in the "no fortification" scenario; (2) shifting the unfortified intake distribution to match this mean while still retaining the original level of variability; and (3) using the probability approach[3] to derive the prevalence of inadequate intakes associated with the new fortified micronutrient intake distribution (**Fig. 1**). We assessed intake inadequacy using the harmonized average requirements (ARs) of Allen et al.[15] for most of the micronutrients (**Fig. S14**); for iron and zinc, the harmonized requirements guided the parameterization of the country-specific ARs developed by Passarelli et al. (2024)[1] to account for factors that inhibit or enhance the absorption of these micronutrients (i.e., phytate intakes and non-dairy animal-source food intakes) (**Fig. S15**). The variability of these requirements also followed the assumptions of Passarelli et al. (2024)[1] (**Table S1**) with the exception of iron. In this analysis, we assumed a log-normal distribution for iron requirements for women of reproductive age (15-49 yr), using a coefficient of variation of 0.44[16] to account for the skewed distribution of iron requirements in this population. As a result, our estimates of the inadequate iron intakes under the "no fortification" scenario differ slightly from those

originally estimated by Passarelli et al. (2024)[1] (**Fig. 2**). These operations were implemented using the *shift_dist()* and *sev()* functions in the *nutriR* package,[4] which was developed to support Passarelli et al. (2022)[2] and Passarelli et al. (2024).[1]

To assess the risk that food fortification might cause adverse health effects from excess micronutrient intakes, we estimated the percent of each subpopulation exceeding the tolerable upper level of intake (ULs) (**Fig. 1**) for the seven micronutrients with harmonized ULs recommended by Allen et al.[15] (**Table S1; Figs. S14 & S15**). Vitamin A is excluded because its intakes are in RAE and its ULs are in retinol. Niacin is excluded because the ULs pertain only to nicotinic acid from supplements or food fortification[15] and our intake distributions characterize all forms of niacin. The harmonized ULs are specified for 13 (5 x 2 sexes + 3 child groups) age-sex groups and were aligned with the 34 age-sex groups used in our analysis following **Table S5**. The prevalence of excess intakes (i.e., the percent of the population above the UL) was calculated using the *above_ul()* function in the *nutriR* package[4].

We calculated the number of people with inadequate or excess intakes using estimates of population size for each country, sex, and age group from the World Bank.[17] We used estimates for 2018, when the global population was approximately 7.57 billion people, as this is the year for which usual dietary intakes were modelled by Passarelli et al. (2024).[1]

Detailed methods and data sources for estimating costs of implementing the fortification scenarios

Micronutrient premix costs

For each country, food vehicle, and fortification scenario, we populated a premix cost calculator, coded in Stata 18, with data on the micronutrient(s), micronutrient level, micronutrient compound, and activity level (i.e., the proportion of micronutrient in the micronutrient compound) to estimate the cost of the micronutrient premix per metric ton (tonne) of food vehicle. Specifically, for the "current fortification" and "improved fortification" scenarios, for each micronutrient listed in the current fortification standard for a specific food vehicle, we used the most recent GFDx data on the mandated micronutrient level at point of fortification or importation and the mandated micronutrient compound. Where a country's fortification standard allowed for more than one micronutrient compound, for iodine we selected potassium iodate for low- and lower-middle-income countries and potassium iodide for upper-middle and high-income countries as the compound for salt fortification. For other micronutrients we (1) selected the compound for which we had price information, and where we had price information for more than one allowable compound in the standard, we (2) selected the lowest-cost compound (generally ferrous sulfate or ferrous fumarate as the iron compound, thiamin mononitrate as the thiamin compound, and zinc oxide as the zinc compound). For the three "aligned" scenarios, data sources to inform micronutrients, micronutrient levels, and micronutrient compounds were as described in section 2.2.2, above. For all scenarios, estimates of global micronutrient compound

and excipient (where relevant) prices as well as compound activity levels were informed by consultations with industry experts in 2023 and 2024. In alignment with the estimates of the prevalence of inadequate micronutrient intakes, we excluded fluoride and vitamin D from all premix cost estimates.

For the special case of rice, we assumed fortified rice kernels (FRK) would be produced via hot extrusion with FRK mixed with unfortified rice at a blending ratio of 1:100[18(p13)]. The cost of FRK accounted for the cost of premix (estimated using the data sources and premix cost calculator described above) in addition to other FRK ingredients (broken rice flour, emulsifier), and manufacturing costs. Broken rice flour and emulsifier prices were based on estimates in the WFP Handbook for the Production of Extruded Fortified Rice Kernels[19] and manufacturing costs, including annualized equipment costs, were assumed to be approximately equal to premix and non-premix ingredient costs (that is, the cost of premix plus other ingredient costs per tonne of FRK were doubled to estimate the total cost of FRK).

We adjusted estimates of the cost of premix per tonne for international shipping, taxes and duties, and domestic transport, storage and handling. Based on input from in-country stakeholders in Malawi (2022) and Ethiopia (2021), for low- and lower-middle-income countries, we added 10% to the cost of the micronutrient premix to account for international shipping. We assumed micronutrient premix would be produced domestically in upper-middle and high-income countries and therefore did not include international shipping costs. In the absence of comprehensive data on country-specific taxes and duties levied against micronutrient premixes, we added 8% to the cost of micronutrient premix across the board to account for duties and taxes. Finally, we added 5% to the cost of the micronutrient premix to account for domestic transport, storage, and handling costs.

To estimate total premix costs, we multiplied estimates of premix cost per tonne by the estimated annual quantity of fortified food vehicle in the food system. For each scenario, an estimate of the annual quantity of fortified food in the food system was calculated as the average daily per capita food intake/availability from GFDx multiplied by the population of the country in 2023 according to the World Population Prospects.

Industry-related costs

To estimate industry-related costs, we first generated a rough estimate of the number of domestic industrial processing facilities for each food vehicle in each country based on the estimated quantity of domestically processed fortifiable food vehicle in the food system divided by the assumed processing capacity of an industrial-scale facility. The quantity of domestically processed fortifiable food vehicle in the food system was estimated as the annual quantity of fortifiable food vehicle in the food system multiplied by the estimated proportion of the food vehicle domestically processed (vs imported). For all food vehicles except salt, the proportion

domestically processed was based on the most recent estimate available from the Food and Agriculture Organization (FAO) Supply Utilization Accounts[20]. In the absence of global data on salt imports and exports, we assumed all salt was domestically refined. Assumed processing capacities of industrial scale facilities were based on the categories (which are specific to country income level and food vehicle) suggested by USAID[21] (USAID, 2022). To account for wide variation in compliance with mandatory standards in the "current fortification" and "aligned standards" scenarios, we also estimated the number of domestic industrial processing facilities engaged in fortification by multiplying the number of domestic industrial processing facilities by the percent compliance and rounding up. For example, if a country had five wheat flour refineries and current compliance was 50%, we estimated that three wheat flour refineries were engaged in fortification. For all other scenarios, we assumed all domestic industrial processing facilities were engaged in fortification.

Then, for each domestic industrial processing facility engaged in fortification, we estimated annual industry-related fortification costs as the sum of annualized fortification equipment costs, annualized quality assurance/quality control (QA/QC) equipment costs, QA/QC supply costs, labor costs for fortification and QA/QC, training costs, and management, overhead, and administration costs. Fortification equipment costs were based on Fiedler & Afidra (2010)[22], Fiedler & Macdonald (2009)[23], and interviews with industry representatives in Ethiopia and Malawi in 2022. QA/QC equipment and supply costs were based on prices in the UNICEF Supply Catalog. Fortification, and QA/QC equipment costs were annualized; we added 7% to the annualized cost to account for equipment maintenance. To estimate full time equivalents (FTEs) for labor related to the fortification process and internal QA/QC, for each food vehicle we placed countries into one of four categories based on quartiles of the quantity of the food vehicle assumed to be fortified at each processing facility. We then assumed 0.2, 0.5, 0.8, and 1 FTE for fortification labor and 0.2, 0.5, 0.8, and 1 FTE for QA/QC labor for countries in the first, second, third, and fourth quartiles, respectively. The cost of labor was estimated as the country-specific GDP per employed person[24] multiplied by the country-specific labor share of GDP[25]. Unit costs for annual training at processing facilities were based on interviews with industry representatives in Ethiopia and Malawi in 2022 and scaled up for upper-middle- and high-income countries. Finally, management, overhead, and administrative costs were estimated as 20% of industry-related costs.

Government-related costs

For existing LSFF programs, we estimated annual government-related costs as the sum of annualized monitoring equipment costs; monitoring supply costs; labor costs for industry, import, and commercial monitoring; social marketing costs; training costs; and management, overhead, and administration costs. Monitoring equipment and supply costs were based on prices in the UNICEF Supply Catalog. Monitoring equipment costs were annualized. We assumed two monitoring visits per domestic industrial processing facility and valued the labor for monitoring

visits based on country-specific GDP per employed person[24] multiplied by the country-specific labor share of GDP[25]. Import monitoring, commercial monitoring, government training, and communications costs were estimated based on interviews with government stakeholders in Ethiopia and Malawi in 2022. Import monitoring costs were scaled by both country income group and the volume of imported food vehicle, with each country assigned to one of four categories based on quartiles of the annual quantity of the food vehicle imported into the country. Similarly, commercial monitoring and government training costs were scaled by country income group as well as the total annual quantity of the food vehicle in the food system. Social marketing costs in upper-middle- and high-income countries were assumed to be zero. Government management, overhead, and administrative costs were estimated as 20% of government-related costs. In the "current fortification" and "aligned standards" scenarios, for food vehicles with mandatory standards, we scaled total annual government-related costs down by 50% for food vehicles with lower than 50% compliance. For these two scenarios we also assumed that government-related costs for food vehicles with voluntary standards would be 25% of the cost of mandatory programs (premix and industry-related costs for voluntary programs were estimated as described above without adjustment).

For new LSFF programs (i.e., those added in the "aligned, improved, and expanded" scenario), government-related costs also included annualized planning and launching costs (e.g., industry assessment, development of standards, development of monitoring and evaluation plans, training), informed based on interviews with government stakeholders in Ethiopia and Malawi. Table S10 summarizes each component of premix, industry-related, and government-related costs that were included in the cost estimates, along with data sources and assumptions.

Sensitivity analyses

Because premix costs often account for the majority of LSFF costs,[26] the accuracy of LSFF cost estimates depends on accurate information on the price of micronutrient compounds. While the cost estimates presented here are based on the best available data on global micronutrient compound prices, there are several reasons why these prices might vary, including fluctuations in demand and volume-based pricing. Particularly for the 'aligned, improved, and expanded' scenario, economies of scale in the premix industry could lead to a reduction in micronutrient compound prices. To account for potential variation in premix prices, we ran a sensitivity analysis to estimate the cost of each scenario assuming a 50% decrease in the price of all micronutrient compounds and then assuming a 50% increase in the price of all compounds. We are not aware of any studies, datasets, or other evidence that might inform the potential size of changes in premix prices. Several recently published papers have used 20-30% variation in premix prices for country-level fortification cost analyses.[27–29] However, because we are, in some scenarios, modeling changes in the global demand for premix with the potential to impact global premix supply chains and prices, we opted to model higher variation in premix prices.

For scenarios that use aligned standards (i.e., scenarios 4 and 5), we ran a sensitivity analysis that excluded calcium in the aligned standard for wheat flour. This is because, although it is included in the WHO guideline for wheat flour fortification, the guideline states that it is seldom used and including it should be dependent on country needs.

For scenarios that use current program compliance (i.e., scenarios 1 and 3), we ran a sensitivity analysis that excluded data for the GFDx indicator on the percentage of food that is fortified in compliance with fortification standards when it was based on proxies of fortification quality from market/household assessments that did not specify meeting fortification standards or estimated fortification quality from expert opinion. The excluded values were replaced with imputed values.

Supplemental Results

Cost analysis

Under the improved compliance scenario, the per capita annual cost of wheat flour and rice fortification would more than triple, while the cost of maize flour fortification would increase by ~$0.02 per capita. Improved compliance would increase the annual cost of refined oil and salt fortification by <$0.01 per capita. With aligned standards, the annual cost of wheat flour fortification would increase to $0.36 per capita at current compliance and to $0.82 per capita with improved compliance, while the cost of maize flour fortification would increase to $0.10 and $0.14 per capita, respectively. The cost of rice fortification with aligned standards would increase to $0.14 and $0.45 per capita based on current and improved compliance, and the annual cost of refined oil and salt fortification per capita would change only slightly if standards were aligned with international guidelines. The per capita costs of implementing aligned standards globally (the aligned, improved, and expanded scenario) are estimated at $0.87 for wheat flour fortification, $0.16 for maize flour, $0.39 for rice, $0.06 for oil, and $0.01 for salt. Except for salt, across food vehicles and scenarios, the cost of micronutrient premix represents at least 70% of the total cost (**Table S11**).

Sensitivity analyses

Compound prices

In all scenarios, varying the price of all micronutrient compounds down and up by 50% decreases/increases the global cost of fortification by 44-48% (**Figure S19**). Salt fortification costs are the least sensitive to variation in compound prices, followed by oil and then the fortified grains.

Aligned standard for calcium in wheat flour

The inclusion of calcium in the aligned wheat flour standards is a major cost driver, more than doubling the estimated global annual fortification cost in the “aligned” and “aligned and improved” scenarios and nearly doubling the cost in the “aligned, improved, and expanded”

scenario (**Figure S20**). Similarly, in each of these scenarios, the annual cost per capita of wheat flour fortification is over 200% higher with calcium compared to without it (**Table S9**). Aligning calcium fortification standards in wheat flour prevents only 16.3 million and 34.9 million inadequate calcium intakes relative to not aligning calcium fortification standards in the "aligned standards" and "aligned and improved" scenarios (**Fig. S17A**). The benefits of aligning calcium fortification standards in wheat flour increased to 637.8 million people with prevented inadequate calcium intakes relative to not aligning calcium fortification standards in wheat flour in the "aligned, improved, and expanded" scenario (**Fig. S17A**). The benefits vary by country but are largely small in the "aligned standards" and "aligned and improved" scenarios and modest in the "aligned, improved, and expanded" scenario (**Fig. S17BC**).

Use of proxy values for fortification compliance

In general, the decision to use proxy values for levels of fortification compliance has small impacts on the global results (**Fig. S18**). The impacts are marginal in the scenarios that include improved compliance since the vast majority of fortification values are increased to 90% under this scenario; as a result, whether a proxy value is used or imputed does not matter, because it is generally increased to 90%. Even under the "current fortification" and "aligned standards" scenarios, the impacts are limited. The maximum difference occurs for thiamin in the "aligned standards" scenario, where 2.7% more of the population has inadequate thiamin intakes when imputing proxy values (i.e., when treating proxy values as unknown) (**Fig. S18**). Similarly, using proxy values vs imputed values has little impact on estimated costs (**Figure S21**).

Supplemental Tables

Table S1. Micronutrients that are added to foods through large-scale food fortification represented in the Global Fortification Data Exchange (GFDx) data.

Micronutrient	Food vehicle(s)	AR source**	AR CV	AR shape	UL source
Vitamins					
Vitamin D*	Rice, wheat flour, maize flour, oil	IOM	0.1	Normal	IOM
Vitamin A	Rice, wheat flour, maize flour, oil	EFSA	0.1	Normal	EFSA***
Vitamin B6 (pyridoxine)	Rice, wheat flour, maize flour	EFSA	0.1	Normal	EFSA
Vitamin B12 (cobalamin)	Rice, wheat flour, maize flour	IOM	0.25	Normal	
Folate (vitamin B9)	Rice, wheat flour, maize flour	EFSA	0.1	Normal	
Niacin (vitamin B3)	Rice, wheat flour, maize flour	IOM	0.1	Normal	EFSA***
Riboflavin (vitamin B2)	Rice, wheat flour, maize flour	EFSA	0.1	Normal	
Thiamin (vitamin B1)	Rice, wheat flour, maize flour	IOM	0.1	Normal	
Vitamin E	Rice, oil	IOM	0.1	Normal	EFSA
Minerals					
Iron	Rice, wheat flour, maize flour, salt	EFSA	0.1	Log-normal	IOM
Calcium	Rice, wheat flour, maize flour	EFSA	0.1	Normal	IOM
Zinc	Rice, wheat flour, maize flour	EFSA	0.1	Normal	EFSA
Selenium	Rice, wheat flour	IOM	0.1	Normal	EFSA
Iodine	Salt	IOM	0.1	Normal	EFSA
Fluoride*	Salt	EFSA	0.1	Normal	EFSA

* Vitamin D and fluoride are not included in the present analysis.

** The source of average requirements (AR) and their coefficients of variation (CV) used to calculate the prevalence of intake inadequacy as recommended by Allen et al. (2020): IOM=US Institute of Medicine and EFSA=European Food Safety Authority.

*** Excluded from analysis of excess intakes because units of upper limits (ULs) and intakes are not aligned.

Table S2. Overview of scenarios modelled to estimate the prevalence of inadequate micronutrient[1] intakes and potential impacts of improving food fortification programs.

Scenario	Fortification standards[2]	Compliance	Notes
**1. No fortification** *(baseline)*	Not applicable	Not applicable	This scenario does not include additional micronutrient intakes from fortified foods.
**2. Current fortification** *(current standards, current compliance)*	Current[3]	Current[3]	In countries with a fortification standard for one or more of the five foods[4], this scenario reflects additional micronutrient intakes based on retaining the current standard for each food.<br><br>In countries with no fortification standard for a specific food, this scenario reflects no additional micronutrient intakes from that food.
**3. Improved compliance** *(current standards, improved compliance)*	Current[3]	90%[5]	Same as scenario #2
**4. Aligned standards** *(aligned standards, current compliance)*	Aligned with international fortification guidelines[6]	Current[3]	In countries with a fortification standard for one or more of the five foods[4], this scenario reflects additional micronutrient intakes based on aligning the standard with international guidelines for each food (in terms of type and amount of micronutrient(s) to add).<br><br>In countries with no fortification standard for a specific food, this scenario reflects no additional micronutrient intakes from that food.
**5. Aligned and improved** *(aligned standards, improved compliance)*	Aligned with international	90%[5]	Same as scenario #4

	fortification guidelines[6]		
**6. Aligned, improved and expanded** *(aligned standards, improved compliance, expanded to countries based on need and availability of an appropriate food)*	Aligned with international fortification guidelines[6]	90%[5]	In all countries, this scenario reflects additional micronutrient intakes based on aligning the standard with international guidelines for all foods that deliver specific micronutrients for which the country-level prevalence of inadequate intakes in no fortification scenario was >20% and an appropriate food was available (based on food intake/availability and proportion industrially processed)

[1] Micronutrients assessed included vitamin E, riboflavin (B2), folate (B9), vitamin B6 (pyridoxine), vitamin A (RAE), vitamin B12 (cobalamin), thiamin (vitamin B1), niacin (vitamin B3), iodine, calcium, iron, zinc, selenium

[2] Included both mandatory and voluntary fortification standards

[3] As reported in GFDx; for compliance [i.e., indicator proportion of food that is fortified (compliance by product volumes)], estimated if data were missing

[4] Fortified foods included were salt, oil, rice, wheat flour, and/or maize flour

[5] Or as reported in the GFDx if current compliance was higher than 90%

[6] Estimated if no existing guideline

Table S3. International fortification standards for all food vehicles except salt. Per capita consumption reflects the country-wide average.

Nutrient (mg /kg)	Per capita consumption (g/day)				
	**Any**	**<75**	**75-149**	**150-300**	**>300**
Wheat flour (WHO 2022)					
Vitamin B12	----	0.04	0.02	0.01	0.008
Vitamin A	----	5.9	3	1.5	1
Folate	----	5	2.6	1.3	1
Riboflavin	----	2	2	2	2
Vitamin B6	----	2	2	2	2
Thiamin	----	3	3	3	3
Zinc	----	95	55	40	30
Iron	----	40	40	20	15
Niacin	----	40	40	40	40
Calcium	----	3125	2112	1250	1250
Maize flour* (WHO 2016)					
Vitamin B12	----	0.04	0.02	0.01	*0.01*
Vitamin A	----	6	3	1.5	*1.5*
Folate	----	5	2.6	1.3	*1.3*
Riboflavin	----	2	2	2	*2*
Vitamin B6	----	6.2	6.2	6.2	*6.2*
Thiamin	----	3.9	3.9	3.9	*3.9*
Iron	----	40	40	20	*20*
Niacin	----	36	36	36	*36*
Zinc	----	95	55	40	*40*
Vitamin B5	----	4.2	4.2	4.2	*4.2*
Rice (WHO 2014)					
Vitamin B12	0.01	----	----	----	----
Folate	1.1	----	----	----	----
Riboflavin	3	----	----	----	----
Vitamin B6	4	----	----	----	----
Thiamin	5	----	----	----	----
Zinc	28.5	----	----	----	----
Niacin	41	----	----	----	----
Iron	42.45	----	----	----	----
Oil					
Vitamin A	18	----	----	----	----

* We assume that the standard for maize flour when consumption is greater than >300 g/day is equivalent to that when consumption is 150-300 g/day. This was not specified in WHO (2016).

Table S4. International fortification standards for salt (WHO 2014).

Nutrient	Intake (g/day)*	Standard (mg/kg)
Salt		
Iodine	<3	65
Iodine	3-4	49
Iodine	4-5	39
Iodine	5-6	33
Iodine	6-7	28
Iodine	7-8	24
Iodine	8-9	22
Iodine	9-10	20
Iodine	10-11	18
Iodine	11-12	16
Iodine	12-13	15
Iodine	>13	14

* This is our interpretation of the intended meaning of these categories. WHO (2014) Table 1 is not clear about recommendations below 3 g/day or above 14 g/day.

Table S5. Alignment of the tolerable upper intake level (UL) age groups from Allen et al. (2020) with the age groups used to estimate inadequate intakes in this paper.

Age group	UL age group
0-4	1-3
5-9	Average of 4-6 and 7-10
10-15	11-14
15-19	15-17
20-24	18-50
25-29	18-50
30-34	18-50
35-39	18-50
40-44	18-50
45-49	18-50
50-54	51-70
55-59	51-70
60-64	51-70
65-69	51-70
70-74	70+
75-79	70+
80+	70+

Table S6. Number of global inadequate intakes by fortification scenario.

Nutrient	Billions of inadequate intakes						Billions of prevented inadequate intakes (relative to no fortification)					Billions of prevented inadequate intakes (relative to current fortification)			
	None	Current	Improved	Aligned	Aligned+	Expanded	Current	Improved	Aligned	Aligned+	Expanded	Improved	Aligned	Aligned+	Expanded
Iodine	3.782	0.473	0.220	0.463	0.214	0.044	3.309	3.562	3.319	3.567	3.738	0.253	0.010	0.258	0.429
Iron	4.693	3.270	2.695	3.239	2.506	1.700	1.423	1.998	1.454	2.187	2.993	0.575	0.031	0.764	1.570
Folate	4.050	3.428	2.780	3.230	1.718	0.838	0.621	1.270	0.820	2.332	3.212	0.648	0.198	1.710	2.590
Zinc	3.480	3.142	2.524	2.921	1.854	0.900	0.338	0.956	0.559	1.626	2.580	0.618	0.221	1.288	2.242
Thiamin	2.235	1.950	1.336	1.809	1.210	0.669	0.284	0.899	0.426	1.025	1.566	0.614	0.141	0.741	1.281
Vitamin A	3.626	3.376	2.295	3.319	1.646	0.927	0.249	1.331	0.307	1.980	2.699	1.082	0.057	1.730	2.449
Riboflavin	4.110	3.873	3.202	3.839	2.921	2.324	0.236	0.908	0.271	1.188	1.786	0.672	0.035	0.952	1.549
Niacin	1.673	1.513	1.254	1.444	1.112	0.828	0.160	0.419	0.229	0.562	0.845	0.259	0.069	0.401	0.685
Vitamin B6	3.880	3.731	3.341	3.660	3.024	1.744	0.149	0.539	0.220	0.856	2.136	0.390	0.071	0.707	1.987
Vitamin B12	2.964	2.841	2.671	2.649	1.969	1.338	0.123	0.293	0.315	0.995	1.626	0.171	0.193	0.873	1.503
Calcium	5.032	4.936	4.496	4.920	4.461	3.859	0.096	0.536	0.112	0.571	1.174	0.440	0.016	0.475	1.078
Vitamin E	5.064	5.063	4.720	5.063	4.720	4.720	0.000	0.344	0.000	0.344	0.344	0.343	0.000	0.343	0.343
Selenium	1.009	1.008	0.997	1.008	0.997	0.997	0.000	0.011	0.000	0.011	0.011	0.011	0.000	0.011	0.011
**Total**	**45.597**	**38.607**	**32.532**	**37.566**	**28.354**	**20.888**	**6.990**	**13.065**	**8.031**	**17.244**	**24.709**	**6.075**	**1.041**	**10.253**	**17.719**

Table S7. Global annual fortification cost per capita.

Scenario	Wheat flour	Maize flour	Rice	Refined oil	Salt	Average total
Current fortification	0.12	0.06	0.10	0.02	0.01	0.18 [0.28]
Improved compliance	0.40	0.08	0.32	0.02	0.01	0.23 [0.33]
Aligned standards	0.36	0.10	0.14	0.02	0.01	0.52 [0.50]
Aligned and improved	0.82	0.14	0.45	0.05	0.01	0.63 [0.53]
Aligned, improved, and expanded	0.87	0.16	0.39	0.06	0.01	1.15 [0.58]

Notes: Global annual cost per capita for each food vehicle calculated as the total annual global cost divided by the sum of the 2023 national populations of each country (UN-DESA, 2024) fortifying the food vehicle. The average total cost per capita (reported as mean [SD]) is the average, among countries fortifying, of the total annual fortification cost per capita by country, where the total annual fortification cost per capita was calculated as the national total annual fortification cost across all five food vehicles divided by the 2023 national population. Costs are expressed in 2021 US dollars. See Table S2, methods, and supplemental methods for details on the scenarios.

Table S8. Countries/territories without sex-age-specific calorie intake estimates and the countries with data from which data was borrowed.

**Without calorie estimates**		**With calorie estimates**	
**ISO3**	**Country/territory**	**ISO3**	**Country/territory**
AFG	Afghanistan	PAK	Pakistan
AND	Andorra	ESP	Spain
BRN	Brunei	MYS	Malaysia
ERI	Eritrea	ETH	Ethiopia
GNQ	Equatorial Guinea	GAB	Gabon
KNA	St. Kitts & Nevis	ATG	Antigua & Barbuda
MHL	Marshall Islands	FSM	Micronesia
NRU	Nauru	FSM	Micronesia
PLW	Palau	FSM	Micronesia
PRK	North Korea	KOR	South Korea
PSE	Palestinian Territories	ISR	Israel
SGP	Singapore	MYS	Malaysia
SOM	Somalia	ETH	Ethiopia
SSD	South Sudan	ETH	Ethiopia
TON	Tonga	FSM	Micronesia
TUV	Tuvalu	FSM	Micronesia
VAT	Vatican City	ITA	Italy

Notes: Sex-age-specific caloric intakes were used to scale population level nutrient-intakes to age-sex specific nutrient intakes.

Table S9. Global annual wheat flour fortification cost per capita, with and without calcium.

**Scenario**	**With calcium**	**Without calcium**
Aligned standards	0.36	0.11
Aligned and improved	0.82	0.26
Aligned, improved, and expanded	0.87	0.26

Notes: Global annual per capita wheat flour fortification costs are calculated as the total annual global cost divided by the sum of the 2023 national populations of each country fortifying wheat flour. Costs are expressed in 2021 US dollars.

Table S10. Components of premix, industry-related, and government-related costs included in the cost estimates and their data sources and assumptions.

Type of cost	Cost component	Sources and assumptions
Premix costs	Micronutrient compounds and excipient	Industry experts
	International shipping	Assumed 10% of premix cost for low- and lower-middle income countries based on input from stakeholders in Malawi and Ethiopia. Assumed 0% for upper-middle- and high-income countries, assuming domestic production of premix.
	Taxes and duties	Assumed 8% of premix cost
	Domestic transport and handling	Assumed 5% of premix cost
Industry-related costs	Fortification equipment	Estimated from Fiedler & Afidra (2010), Fiedler & Macdonald (2009), and interviews with industry in Ethiopia and Malawi
	Quality assurance/quality control (QA/QC) equipment and maintenance	UNICEF Supply Catalog plus 7% of annualized cost for maintenance
	QA/QC supplies	UNICEF Supply Catalog
	Labor for fortification	Full-time equivalent requirements informed by interviews with industry in Ethiopia and Malawi and scaled by quartiles of the quantity of the food vehicle assumed to be fortified at each processing facility. Value of labor estimated as country-specific GDP per employed person multiplied by the country-specific labor share of GDP.
	Labor for QA/QC	Full-time equivalent requirements informed by interviews with industry in Ethiopia and Malawi and scaled by quartiles of the quantity of the food vehicle assumed to be fortified at each processing facility. Value of labor estimated as country-specific GDP per employed person multiplied by the country-specific labor share of GDP.
	Training	Based on interviews with industry representatives in Ethiopia and Malawi and scaled up by 200% for upper-middle- and high-income countries
	Management, overhead, and administration	Assumed 20% of industry-related costs

Government-related costs[1]	Monitoring equipment and maintenance	UNICEF Supply Catalog plus 7% of annualized cost for maintenance
	Monitoring supplies	UNICEF Supply Catalog
	Labor for industry monitoring	Assumed two monitoring visits per domestic industrial processing facility per year, Labor for monitoring visits valued as country-specific GDP per employed person multiplied by the country-specific labor share of GDP.
	Labor for commercial monitoring	Informed by interviews with government stakeholders in Ethiopia and Malawi and scaled by country income group
	Labor for import monitoring	Informed by interviews with government stakeholders in Ethiopia and Malawi and scaled by country income group and the volume of imported food vehicle, with each country assigned to one of four categories based on quartiles of the annual quantity of the food vehicle imported into the country
	Social marketing	Informed by interviews with government stakeholders in Ethiopia and Malawi (assumed zero in middle- and high-income countries)
	Training	Informed by interviews with government stakeholders in Ethiopia and Malawi, scaled by country income group
	Management, overhead, and administration	Assumed 20% of government-related costs

[1]Note that in the "current fortification" and "aligned standards" scenarios, for food vehicles with mandatory standards, total annual government-related costs were scaled down by 50% for food vehicles with lower than 50% compliance. For food vehicles with voluntary standards in the "current fortification" and "aligned standards", we assumed total annual government-related costs would be 25% of mandatory programs.

Table S11. Premix costs as a percent of total cost

Scenario	Wheat flour	Maize flour	Rice	Refined oil	Salt
Current fortification	74 [22]	81 [15]	86 [4]	79 [14]	40 [27]
Improved compliance	77 [20]	83 [15]	88 [7]	80 [13]	40 [27]
Aligned standards	93 [13]	89 [12]	91 [3]	83 [10]	41 [27]
Aligned and improved	95 [11]	90 [12]	92 [5]	84 [10]	41 [27]
Aligned, improved, and expanded	92 [12]	82 [16]	81 [15]	70 [22]	36 [28]

Values presented as mean [SD].

Supplemental Figures

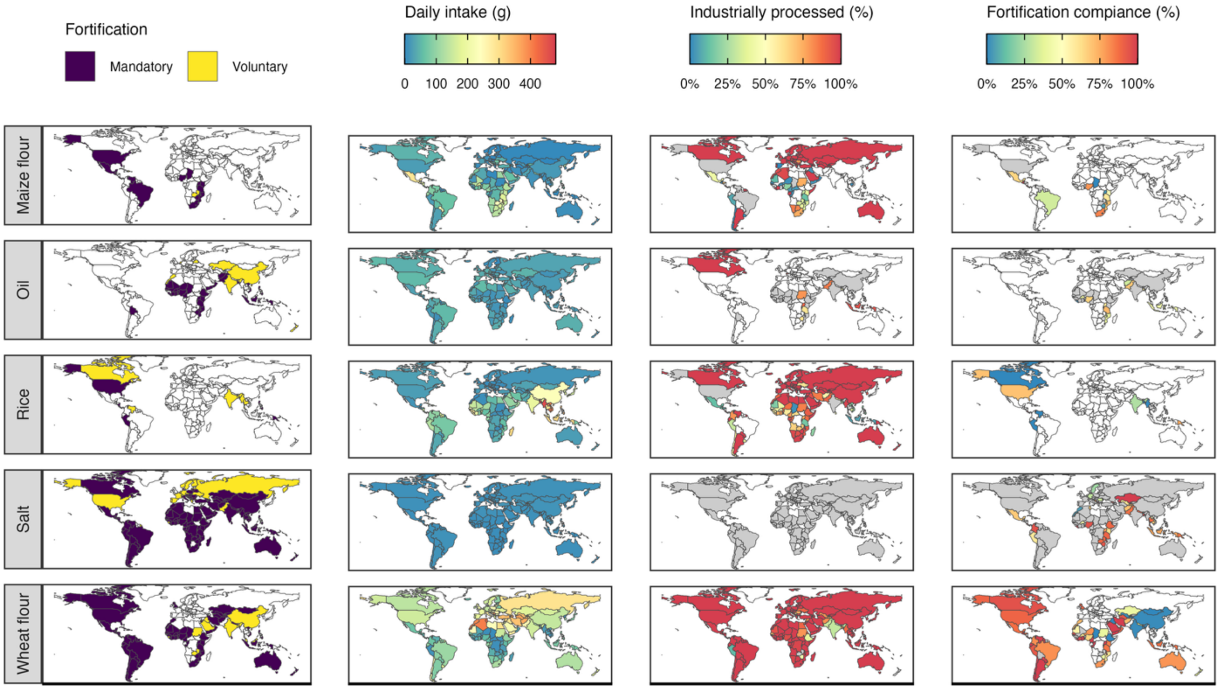

Figure S1. The food vehicle-specific **(A)** type of fortification program, **(B)** daily food intake/availability per capita, **(C)** proportion of food that is industrially processed, and **(D)** proportion of food that is fortified in compliance with fortification standards based on the GFDx (GFDx, 2024). White indicates countries without fortification programs and grey indicates countries with fortification programs that are not completely documented in the GFDx. Missing values are imputed through procedures described in the supplemental methods.

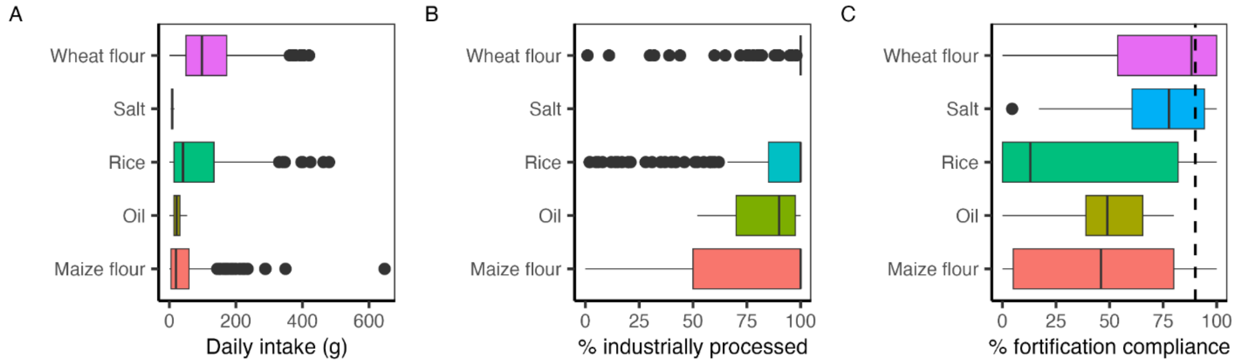

Figure S2. Distribution of data on the **(A)** daily food intake/availability per capita, **(B)** proportion of food that is industrially processed, and **(C)** proportion of food vehicle that is fortified in compliance with fortification standards in the GFDx (GFDx, 2024). In the boxplots, the solid line indicates the median, the box indicates the interquartile range (IQR; 25th to 75th percentiles), the whiskers indicate 1.5 times the IQR, and points indicate outliers.

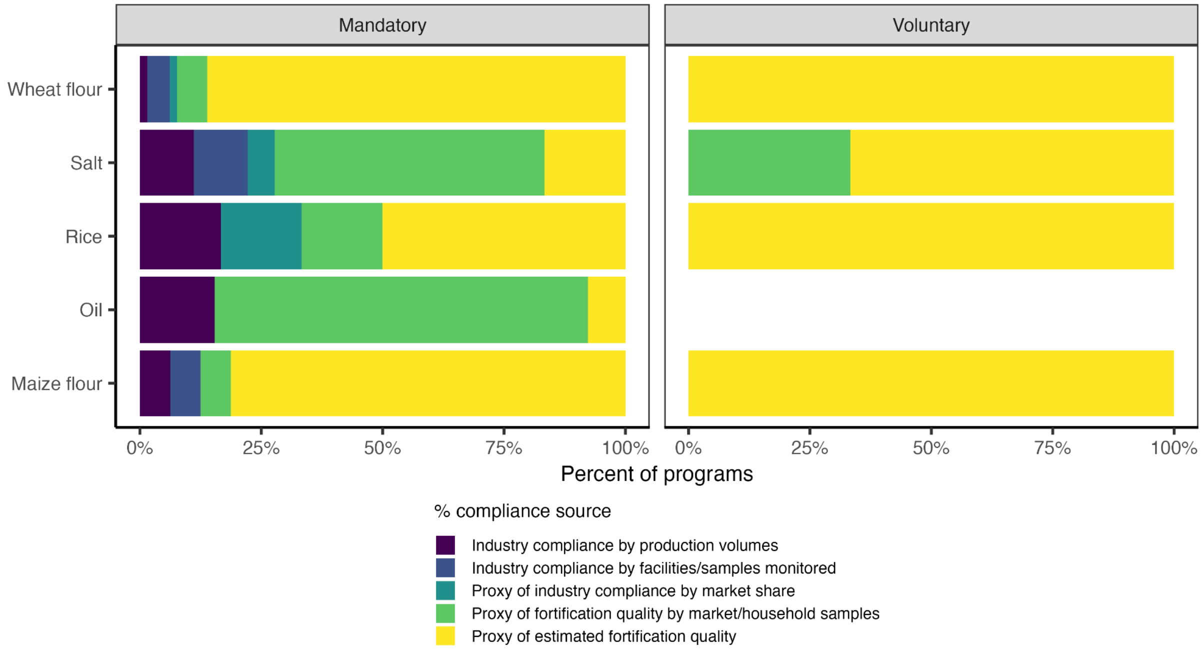

Figure S3. The source of compliance estimates in the GFDx (GFDx, 2024) among mandatory and voluntary fortification programs for each food vehicle. Sources are listed from most reliable (industry compliance by production volumes) to least reliable (proxy of estimated fortification quality).

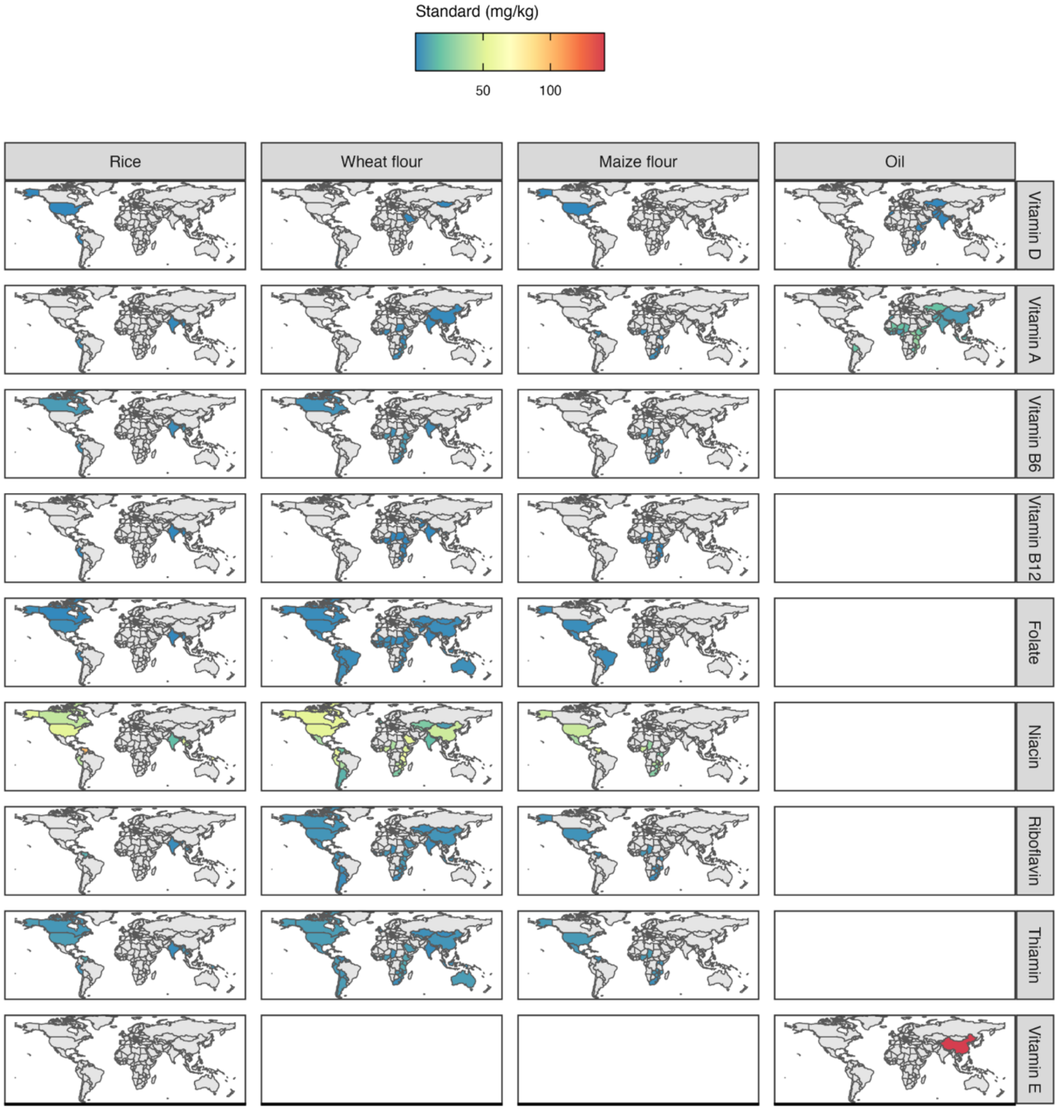

Figure S4. The micronutrient levels in current fortification standards for vitamins for the four food vehicles delivering vitamins (salt is not fortified with vitamins) based on the GFDx data (GFDx, 2024).

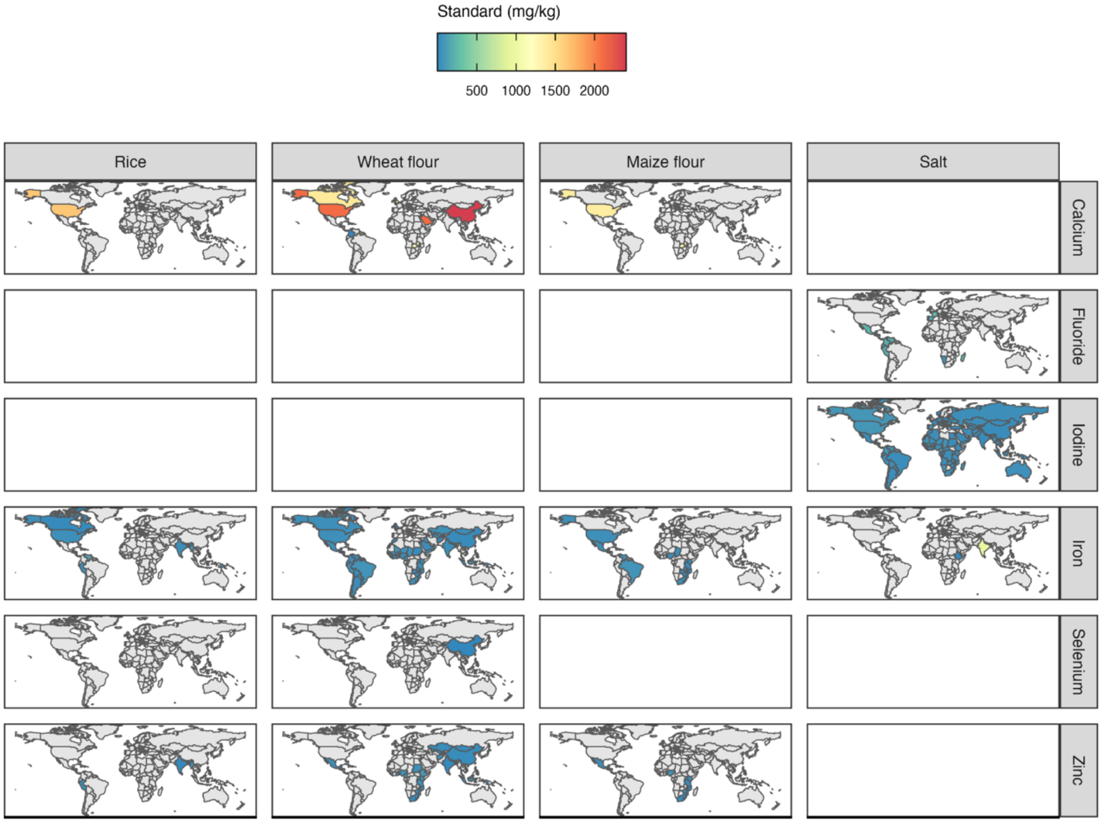

Figure S5. The micronutrient levels in current fortification standards (mg/kg) for minerals for the four food vehicles delivering minerals (oil is not fortified with minerals) based on the GFDx data (GFDx, 2024).

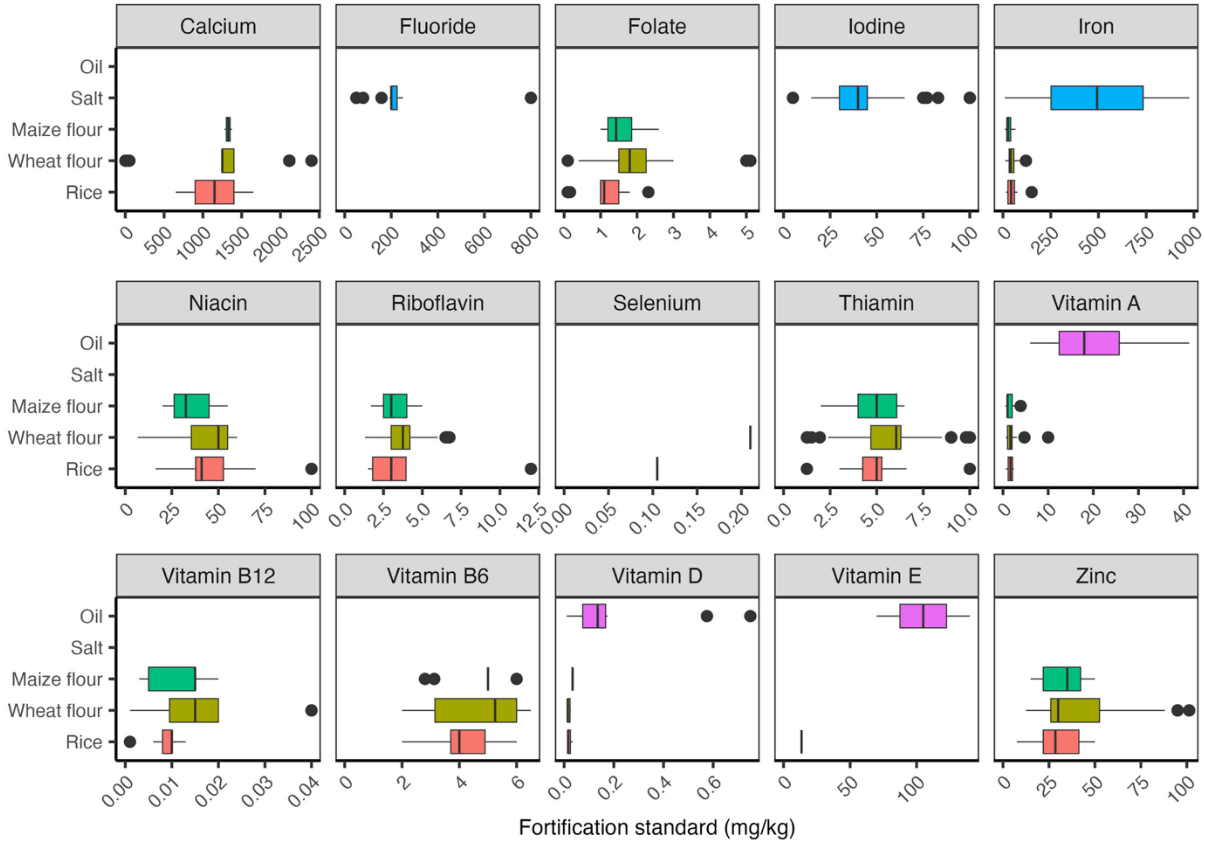

Figure S6. Distribution of micronutrient levels in current fortification standards (mg/kg) in the GFDx (GFDx, 2024). In the boxplots, the solid line indicates the median, the box indicates the interquartile range (IQR; 25th to 75th percentiles), the whiskers indicate 1.5 times the IQR, and points indicate outliers.

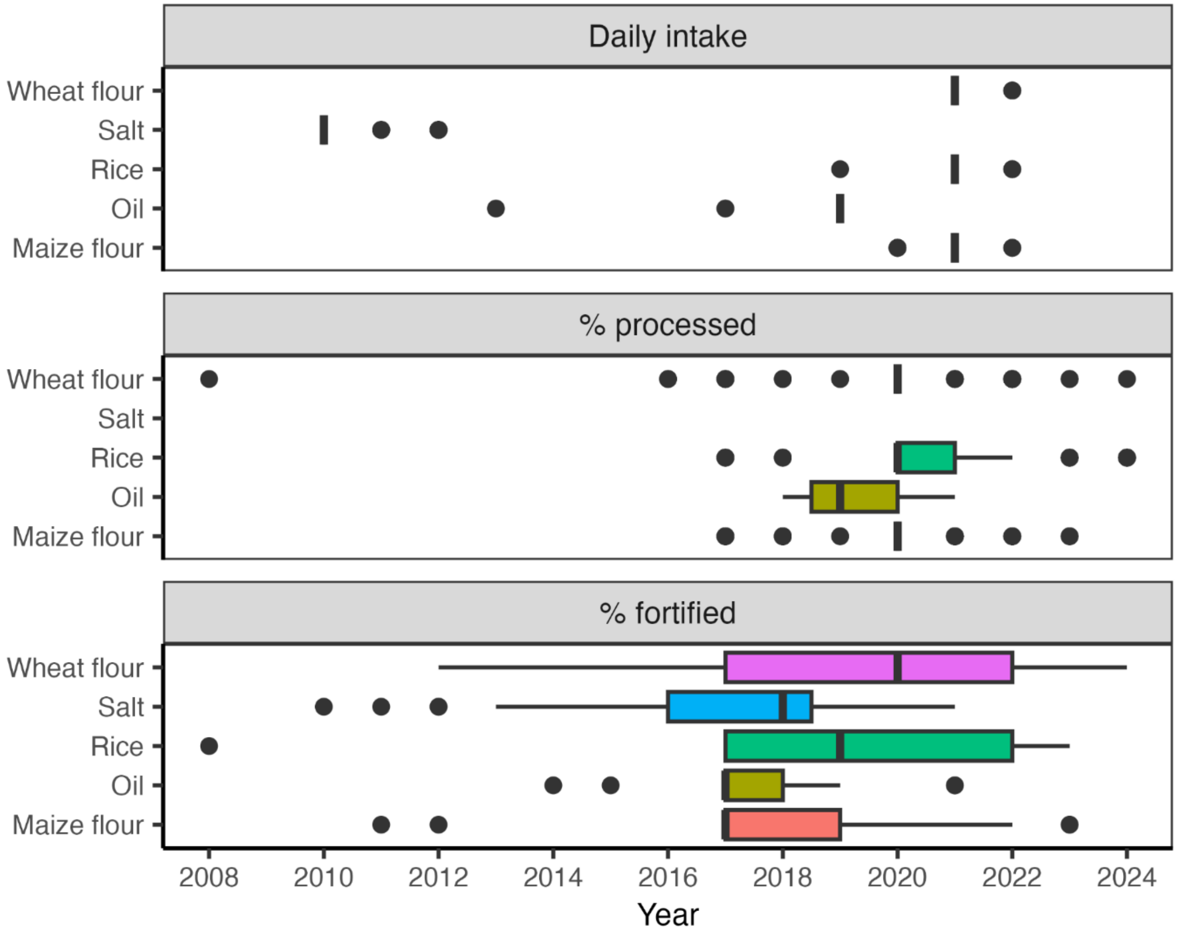

Figure S7. The most recent year with data on the **(A)** daily food intake/availability per capita, **(B)** proportion of food that is industrially processed, and **(C)** proportion food that is fortified in compliance with fortification standards in the GFDx (GFDx, 2024). In the boxplots, the solid line indicates the median, the box indicates the interquartile range (IQR; 25th to 75th percentiles), the whiskers indicate 1.5 times the IQR, and points indicate outliers.

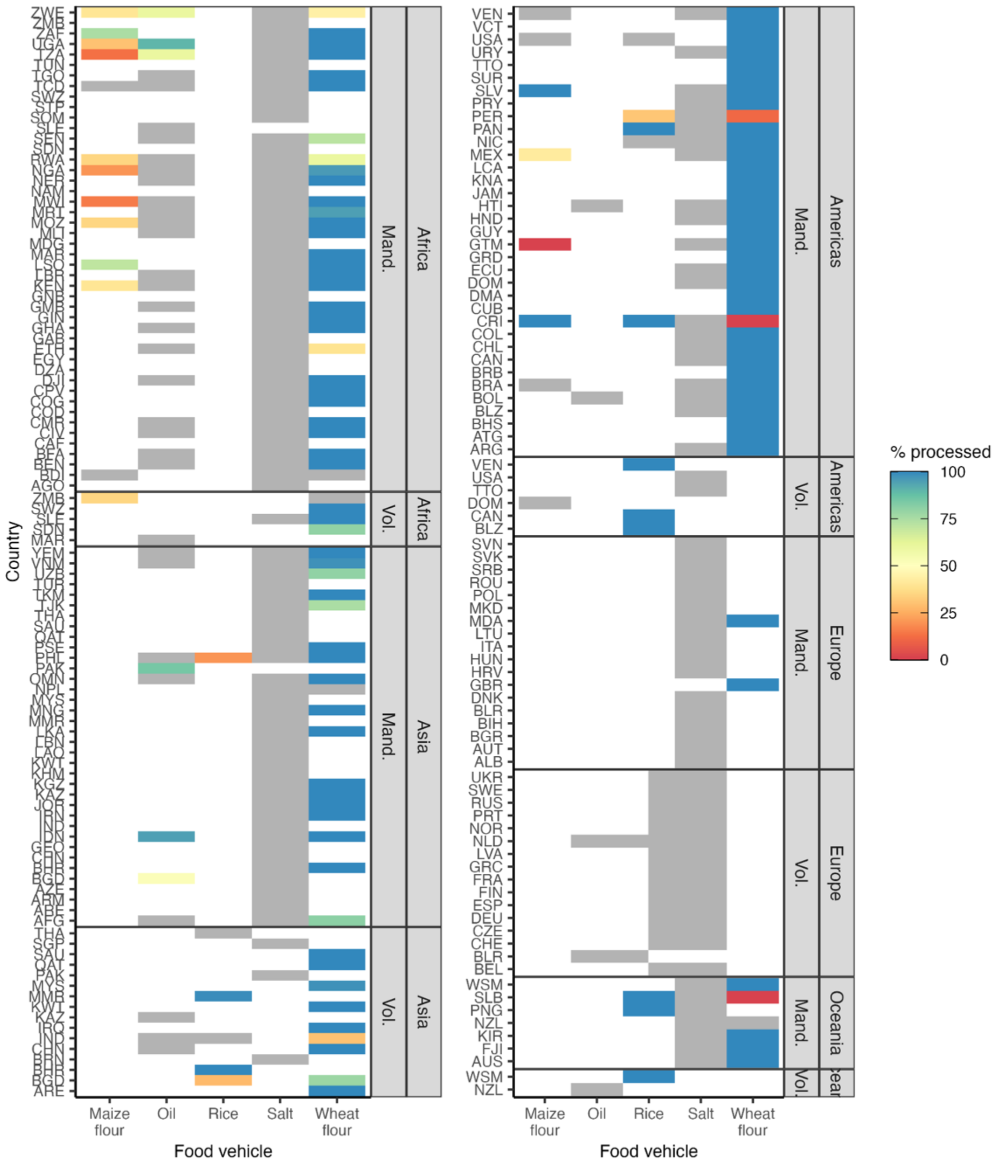

Figure S8. The availability of country-level data on the proportion of food that is industrially processed in the GFDx. Countries are grouped by continent and labeled with their ISO3 code. Colored cells indicate values available within the GFDx. Gray cells indicate missing values that need to be imputed for our analysis. White cells indicate missing values that do not need to be imputed for our analysis given the absence of a relevant fortification program.

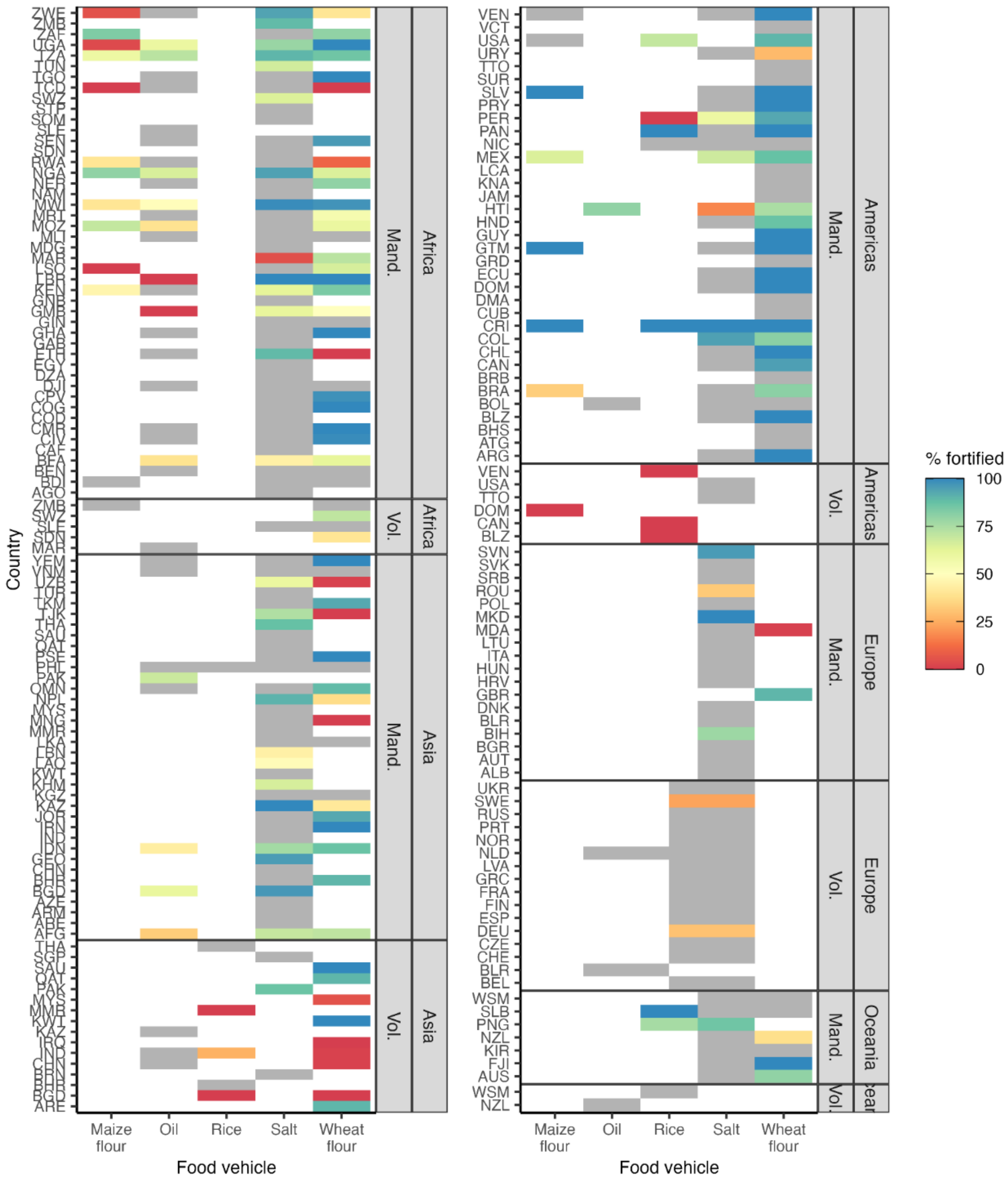

Figure S9. The availability of country-level data on the proportion of food that is fortified in compliance with fortification standards in the GFDx. Countries are grouped by continent and the type of fortification program (mandatory or voluntary) and labeled with their ISO3 code. Colored cells indicate values available within the GFDx. Gray cells indicate missing values that need to be imputed for our analysis. White cells indicate missing values that do not need to be imputed for our analysis given the absence of a relevant fortification program.

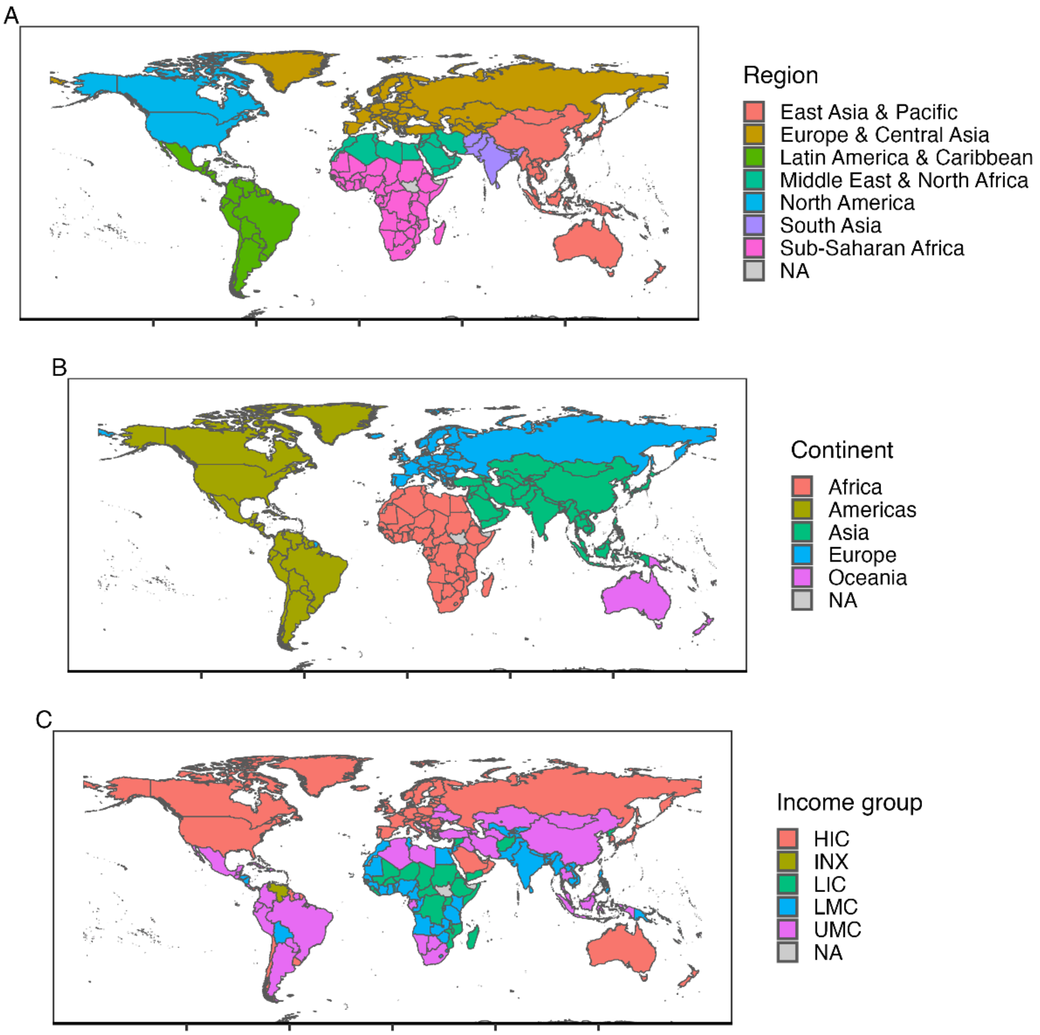

Figure S10. World Bank regions used to guide GFDx imputation.

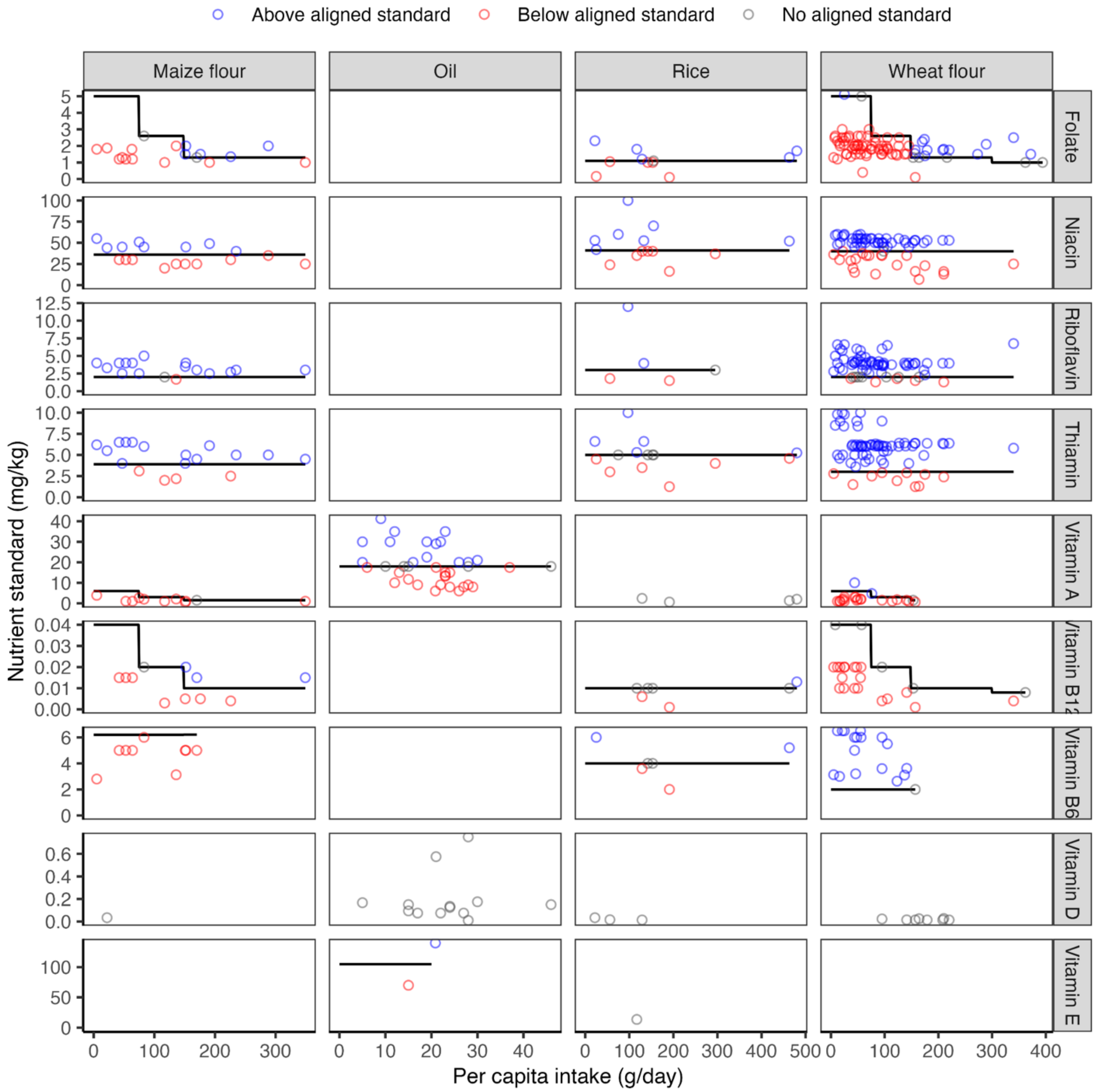

Figure S11. Micronutrient levels in current fortification standards (mg/kg) produced by the aligned standards algorithm (black line) and current fortification standards (points) for vitamins by food vehicle and micronutrient. Each point thus represents the current fortification standard in the country relative to its current average per capita intake of each fortificant. The "aligned standards" scenario uses the current fortification standard when it includes additional micronutrients or micronutrients at levels higher than what was specified in the aligned standards algorithm.

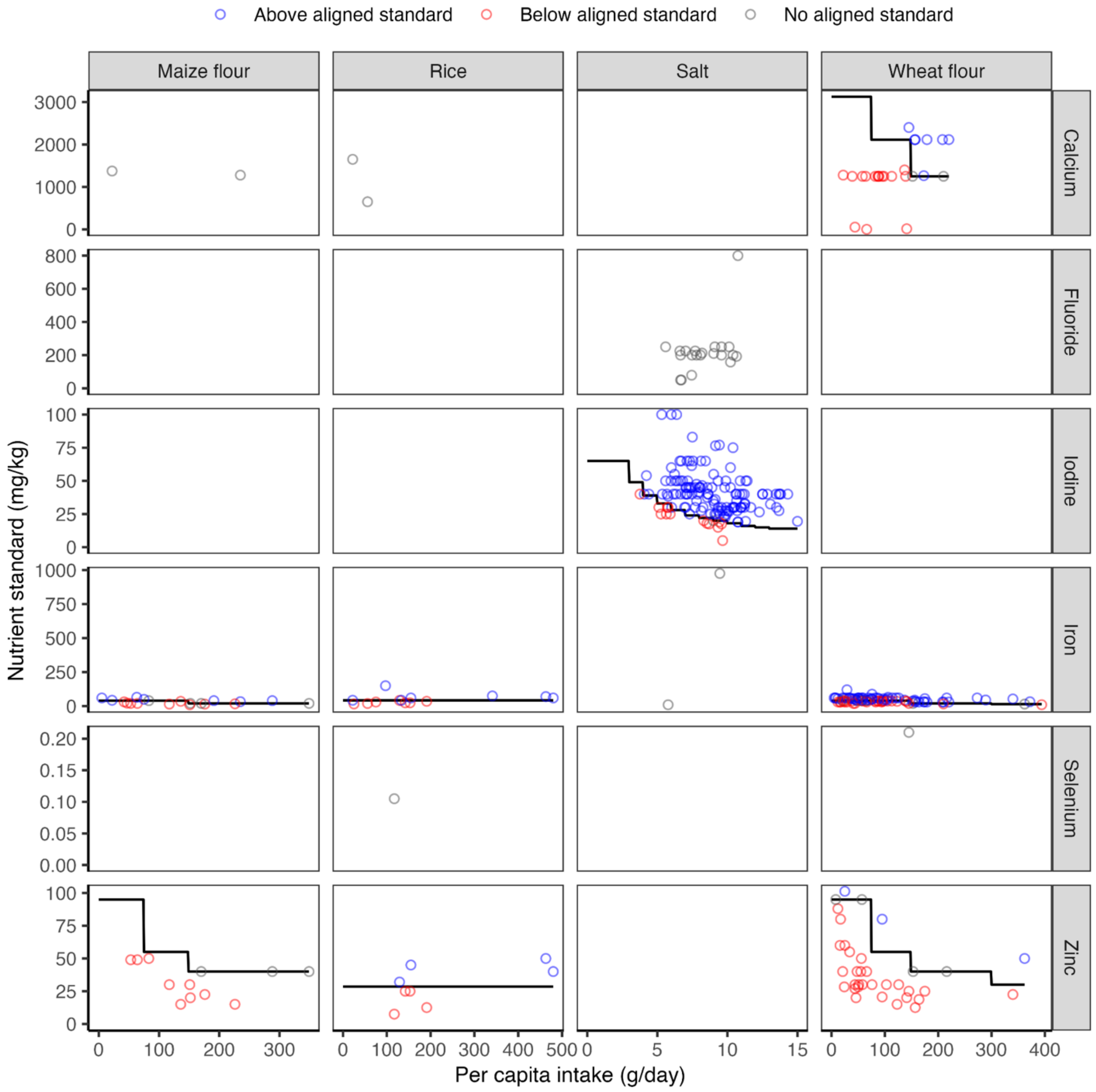

Figure S12. Micronutrient levels in current fortification standards (mg/kg) produced by the aligned standards algorithm (black line) and current fortification standards (points) for minerals by food vehicle and micronutrient. Each point thus represents the current fortification standard in the country relative to its current average per capita intake of each fortificant. The "aligned standards" scenario uses the current fortification standard when it includes additional micronutrients or micronutrients at levels higher than what was specified in the "aligned standards" algorithm.

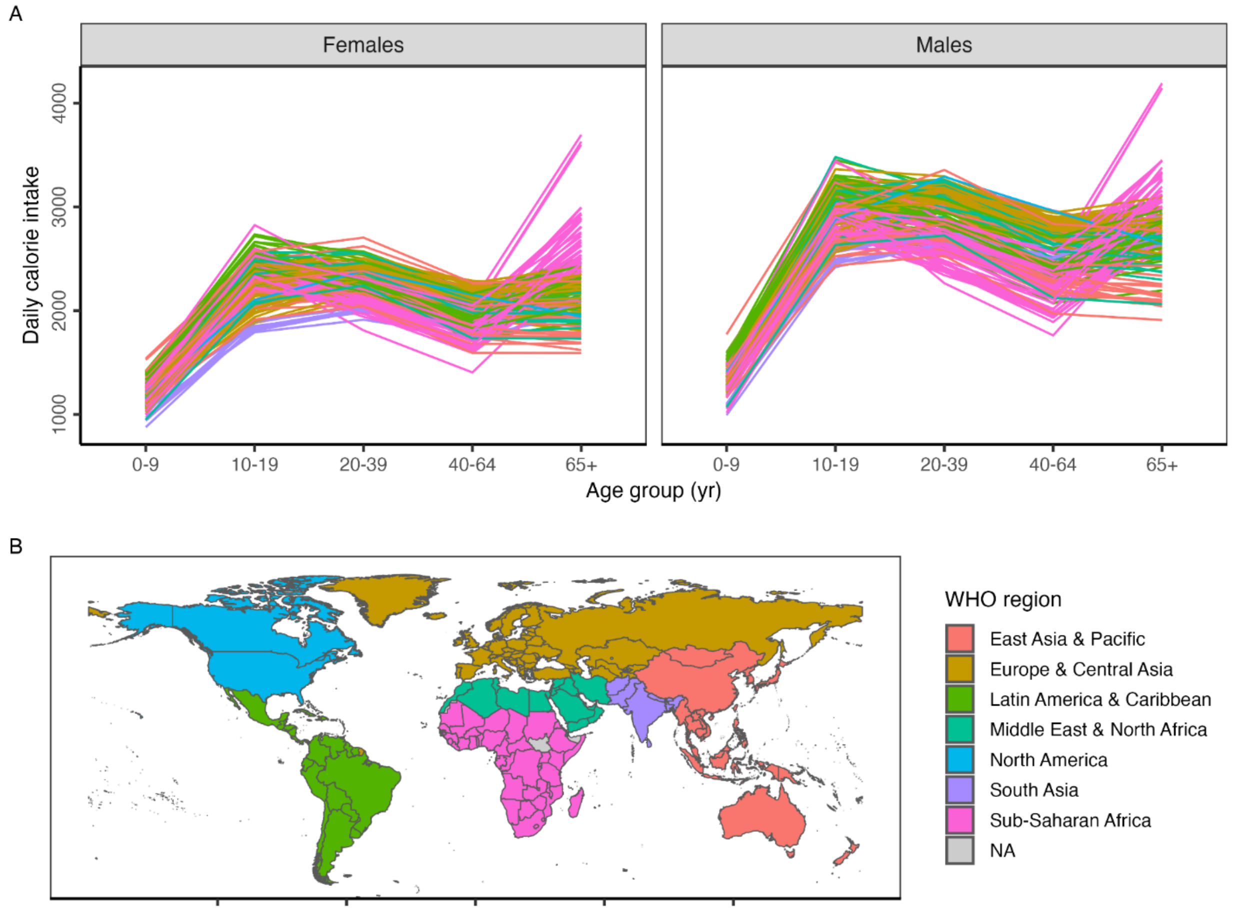

Figure S13. Average calorie intakes by country, sex, and age group based on (Springmann, 2025).

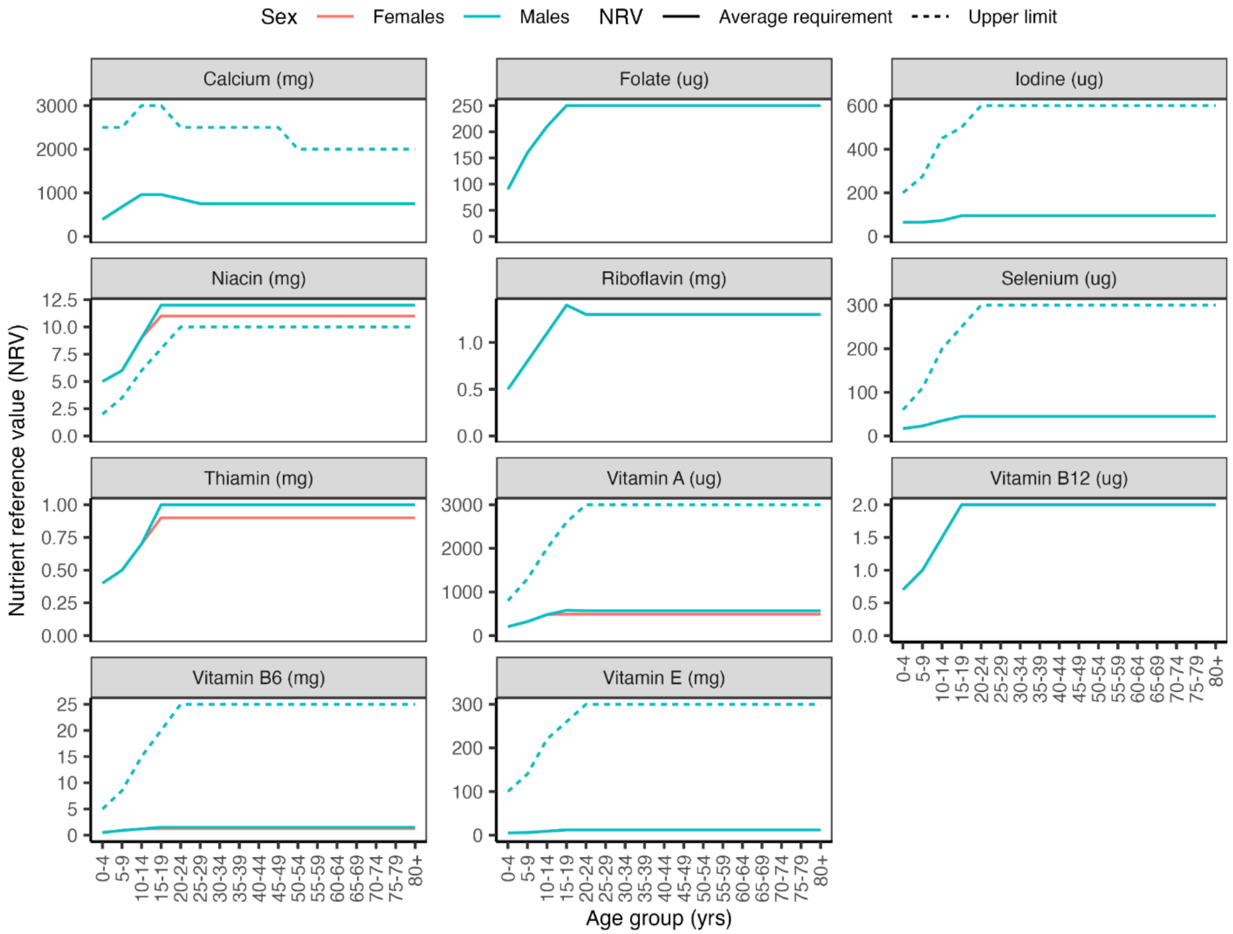

Figure S14. Average requirements (solid lines) and tolerable upper intake levels (ULs, when available; dotted lines) for most of the evaluated micronutrients (iron and zinc are shown in **Figure S15**). Average requirements are identical for males and females for some micronutrients (i.e., calcium, folate, iodine, riboflavin, selenium, vitamin B12, and vitamin E) . ULs have not been specified for folate, riboflavin, thiamin, or vitamin B12. ULs are identical for males and females for the nutrients for which they have been specified. The ULs for niacin only pertain to nicotinic acid from supplements or food fortification, which is why the UL is lower than the average requirement (which pertains to all forms of niacin).

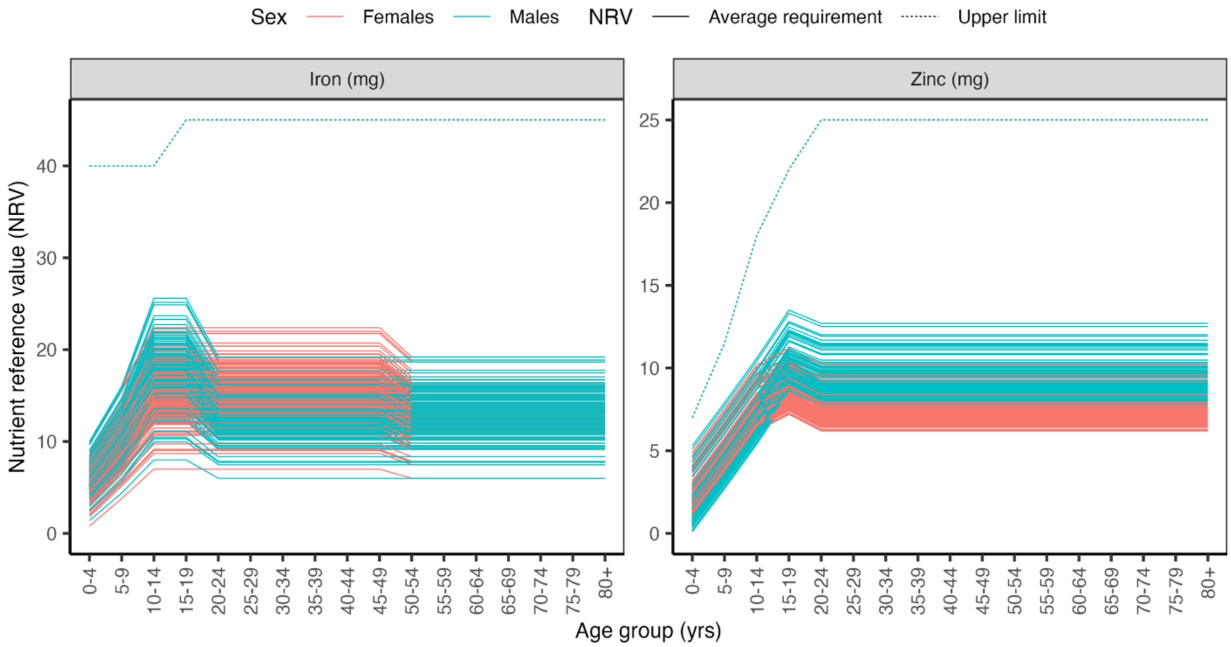

Figure S15. Average requirements (solid lines) and tolerable upper intake levels (dotted lines) for iron and zinc. Average requirements are country-specific based on levels of phytate and non-dairy animal-source food intakes.

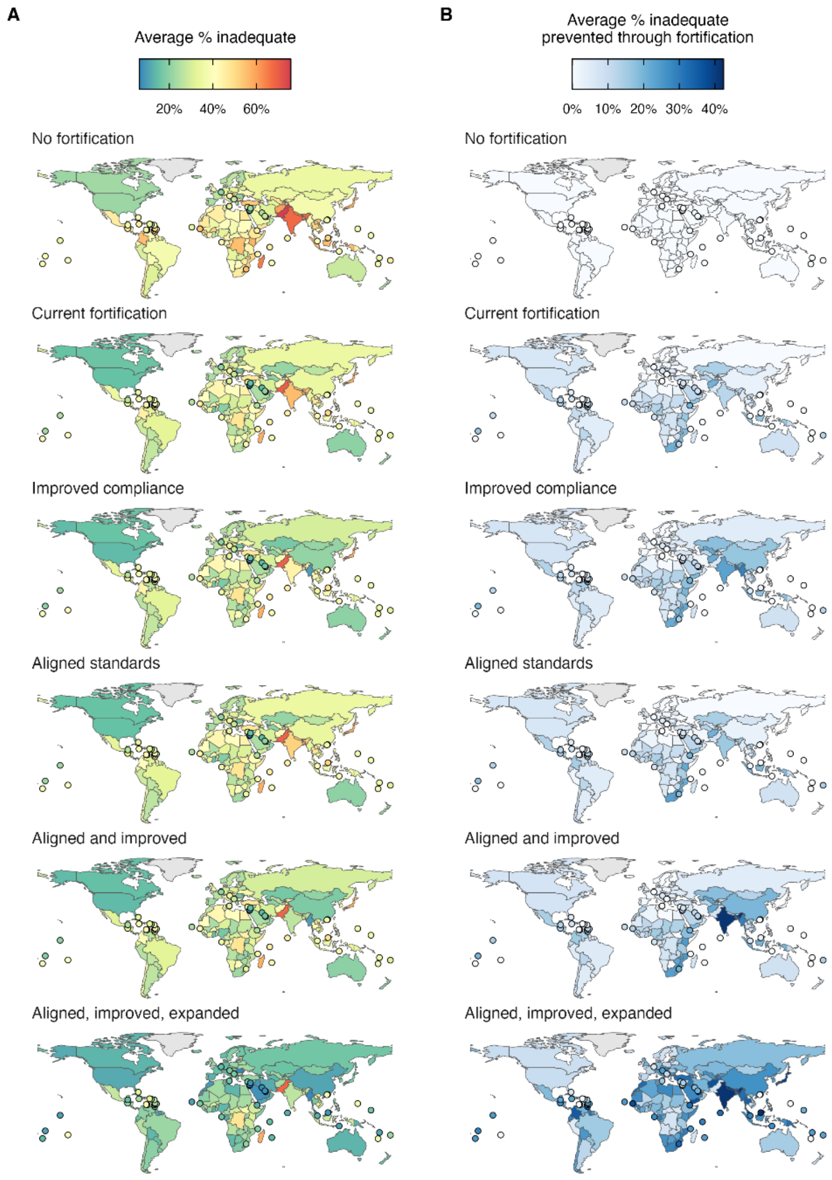

Figure S16. The **(A)** average estimated prevalence of inadequate intakes across the 13 evaluated micronutrients and **(B)** average prevalence of inadequate intakes prevented through fortification (i.e., relative to the "no fortification" scenario) by country and fortification scenario. Countries with land areas less than 25,000 km^2 are shown as points to increase visibility.

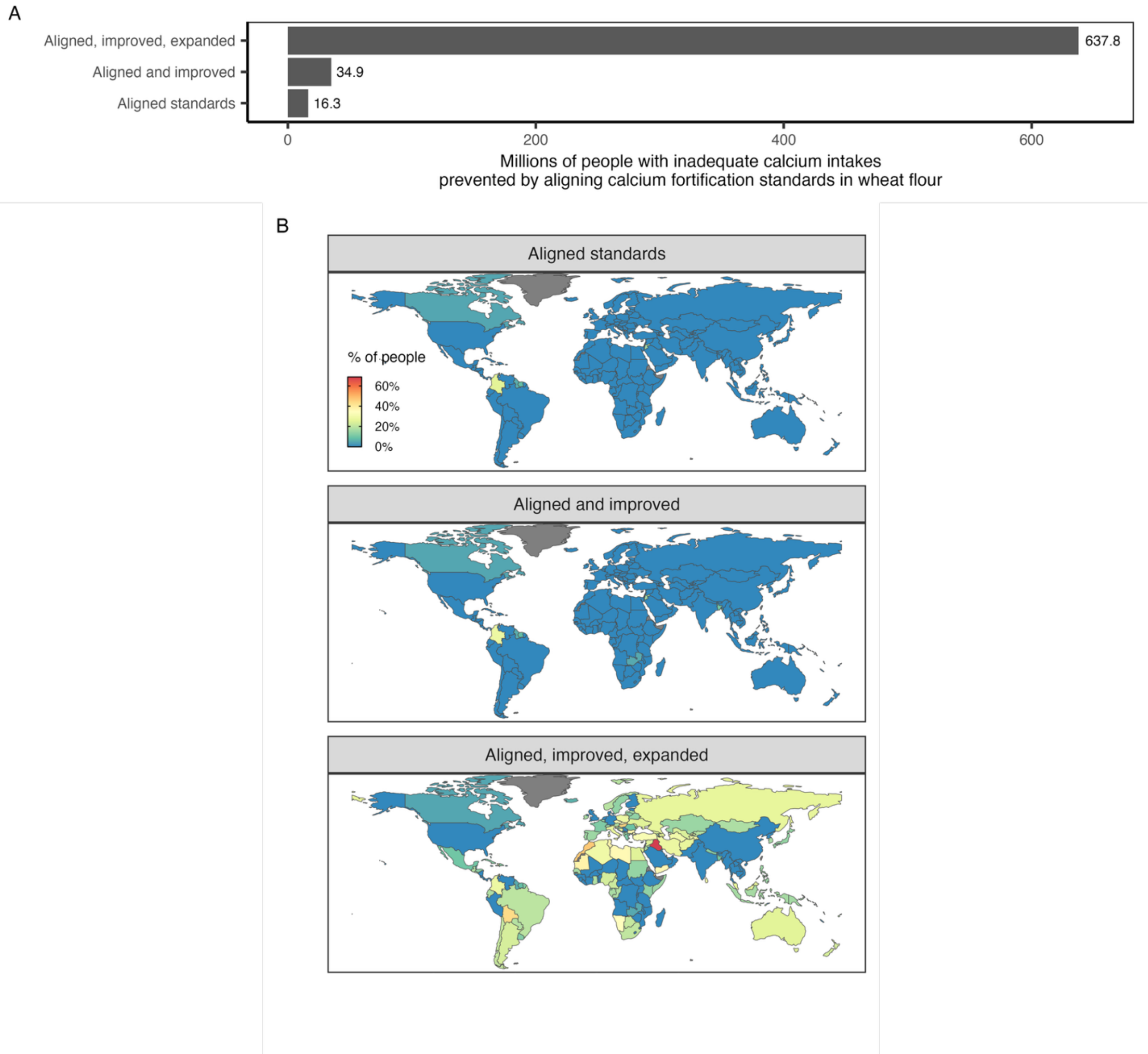

Figure S17. The results of a sensitivity analysis examining the impact of using aligned standards for calcium in wheat flour in the scenarios employing aligned standards. Panel **A** shows the global number of people with inadequate intakes prevented by aligning calcium fortification standards in wheat flour (i.e., how many more inadequate calcium intakes there are when no aligned standards for calcium in wheat flour are used) under scenarios employing aligned standards. Panel **B** shows the percentage of national populations with inadequate intakes prevented by aligning calcium fortification standards in wheat flour.

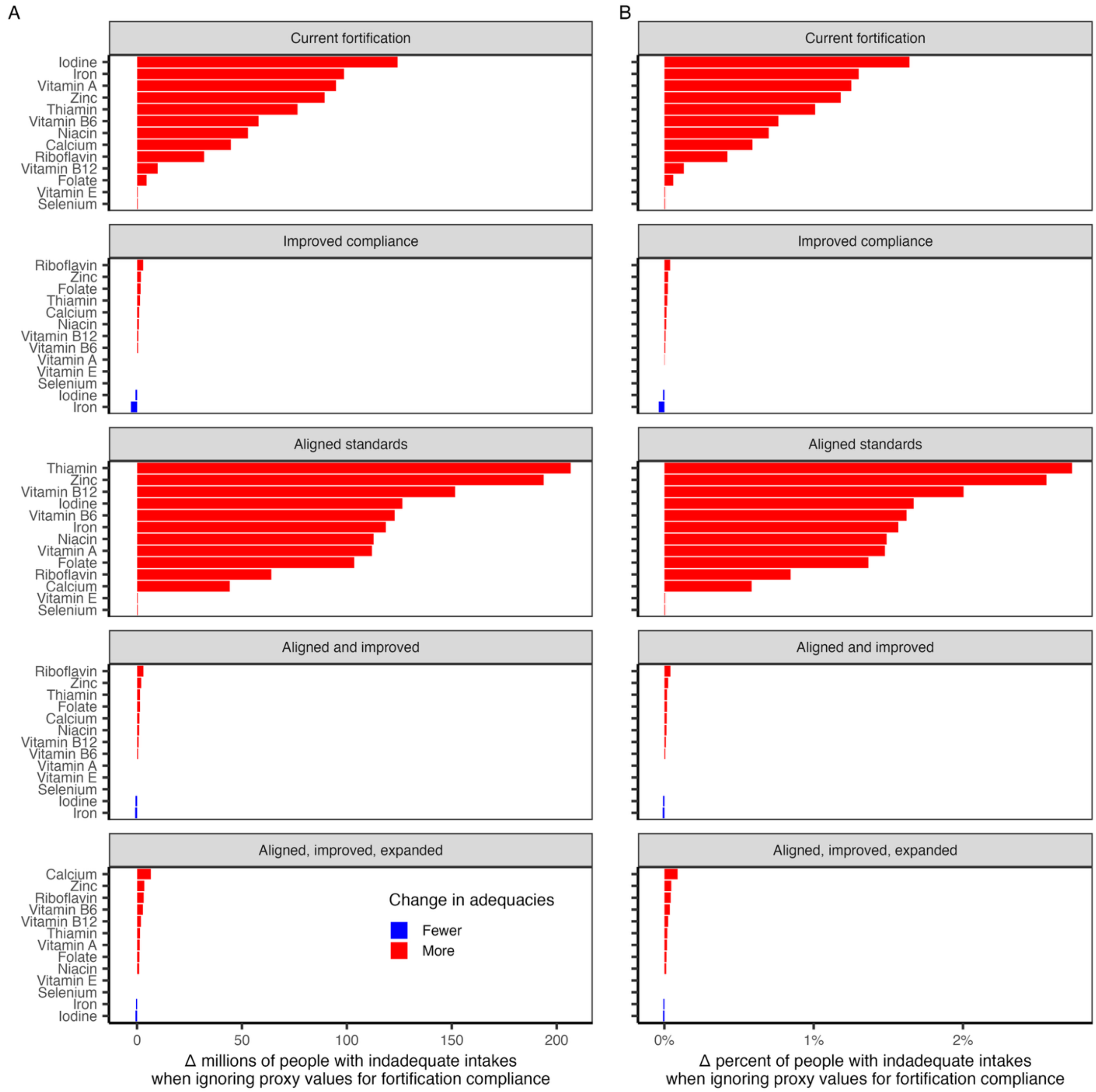

Figure S18. The difference in (A) the number and (B) percent of people with inadequate intakes globally when treating estimates of fortification compliance from proxy sources as unknown and thus requiring imputation (i.e., filling missing values with surrogates). Negative values indicate nutrients for which the prevalence of inadequate intakes decreases when imputing proxy values and positive values indicate nutrients for which the prevalence of inadequate intakes increases when imputing proxy values. The scenarios without improved compliance are most sensitive to this decision because most current fortification compliance values are below the 90% compliance level assumed in the “improved compliance” scenarios. Overall, the decision to use proxy values (i.e., treat them as known and not requiring imputations) has little influence on the global results; the largest impacts represent <3% of the global population.

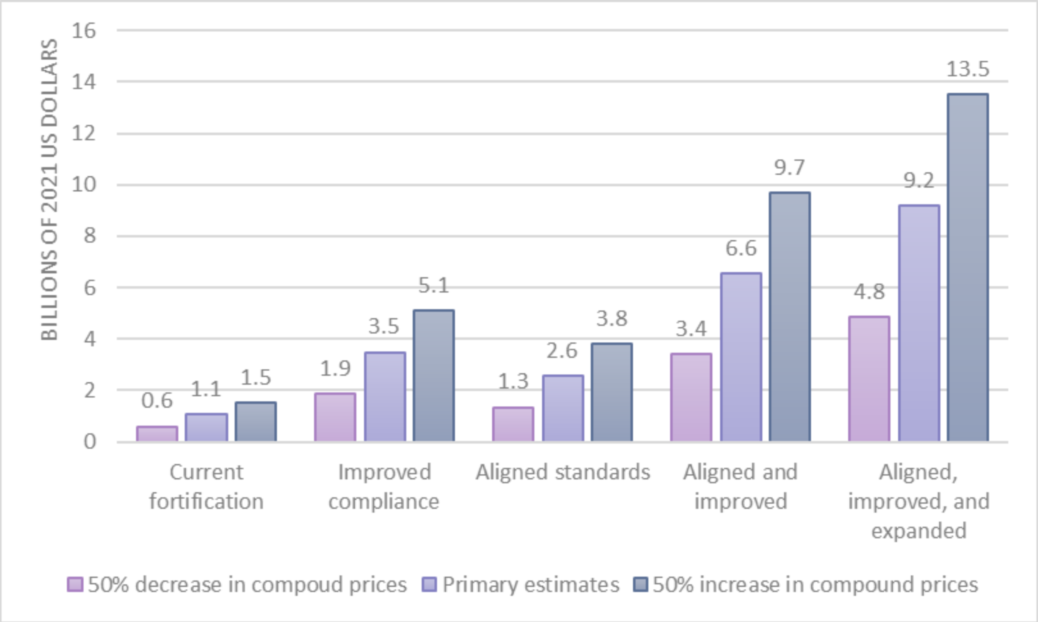

Figure S19. Global annual cost of fortification assuming a 50% decrease in the price of all micronutrient compounds and a 50% increase in the price of all micronutrient compounds, compared to primary estimates (2021 US dollars). Cost estimates include premix, industry-related, and government-related costs for wheat flour, maize flour, rice, refined oil, and salt fortification.

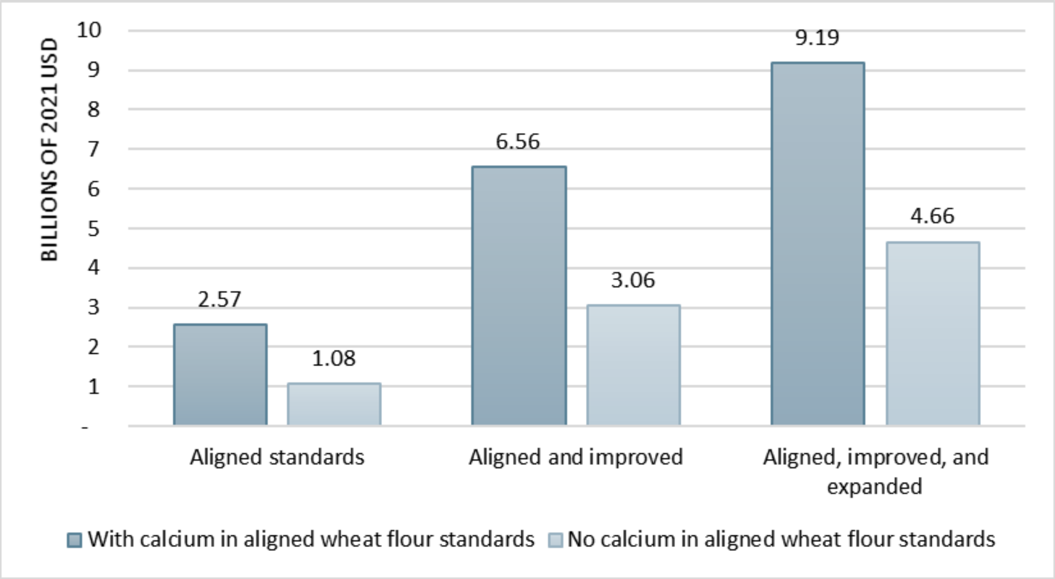

Figure S20. Global annual cost of fortification with standards aligned to international guidelines, with and without the inclusion of calcium in aligned wheat flour standards (2021 US dollars). Cost estimates include premix, industry-related, and government-related costs for wheat flour, maize flour, rice, refined oil, and salt fortification.

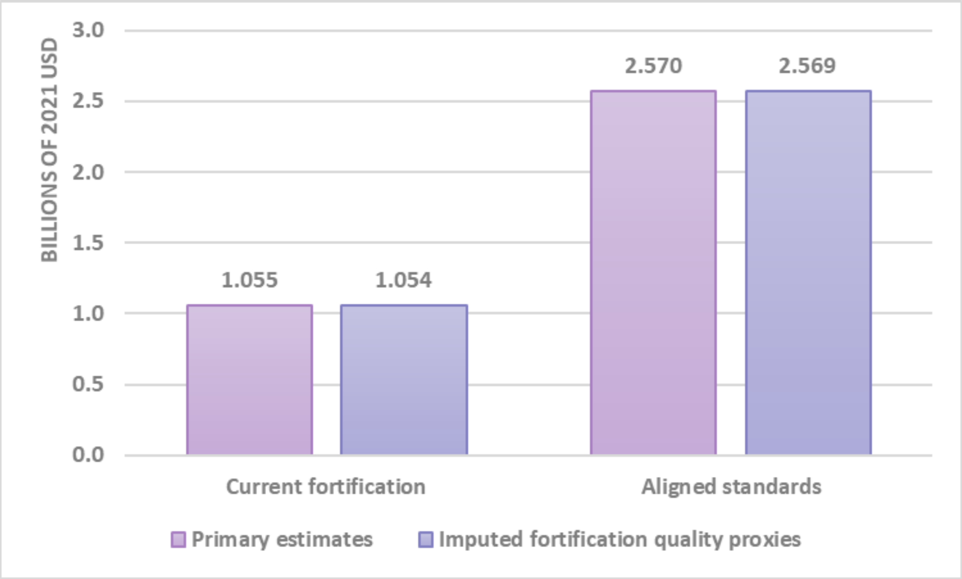

Figure S21. Global annual cost (2021 US dollars) of current fortification and with standards aligned to international guidelines including proxies for fortification quality (primary estimates) and replacing proxies for fortification quality with imputed values (imputed fortification quality proxies). Cost estimates include premix, industry-related, and government-related costs for wheat flour, maize flour, rice, refined oil, and salt fortification.

References

1. Passarelli S, Free CM, Shepon A, Beal T, Batis C, Golden CD. Global estimation of dietary micronutrient inadequacies: a modelling analysis. *The Lancet Global Health*. Published online August 29, 2024. doi:10.1016/S2214-109X(24)00276-6

2. Passarelli S, Free CM, Allen LH, et al. Estimating national and subnational nutrient intake distributions of global diets. *The American Journal of Clinical Nutrition*. 2022;116(2):551-560. doi:10.1093/ajcn/nqac108

3. National Research Council. *Nutrient Adequacy: Assessment Using Food Consumption Surveys*. National Academies Press (US); 1986.

4. Free C, Passarelli S, Allen LH, Beal T, Biltoft-Jensen AP. nutriR: Nutritional intake functions for R. Published online 2022. Accessed February 11, 2025. https://github.com/cfree14/nutriR

5. Food and Agriculture Organization of the United Nations (FAO). FAOSTAT. Accessed January 24, 2025. https://www.fao.org/faostat/en/#data

6. Powles J, Fahimi S, Micha R, et al. Global, regional and national sodium intakes in 1990 and 2010: a systematic analysis of 24 h urinary sodium excretion and dietary surveys worldwide. *BMJ Open*. 2013;3(12):e003733. doi:10.1136/bmjopen-2013-003733

7. Trieu K, Neal B, Hawkes C, et al. Salt Reduction Initiatives around the World – A Systematic Review of Progress towards the Global Target. *PLOS ONE*. 2015;10(7):e0130247. doi:10.1371/journal.pone.0130247

8. Lips P, van Schoor NM, de Jongh RT. Diet, sun, and lifestyle as determinants of vitamin D status. *Annals of the New York Academy of Sciences*. 2014;1317(1):92-98. doi:10.1111/nyas.12443

9. Wessells KR, Manger MS, Tsang BL, Brown KH, McDonald CM. Mandatory large-scale food fortification programmes can reduce the estimated prevalence of inadequate zinc intake by up to 50% globally. *Nat Food*. 2024;5(7):625-637. doi:10.1038/s43016-024-00997-w

10. World Health Organization. *Guideline: Fortification of Wheat Flour with Vitamins and Minerals as a Public Health Strategy*. World Health Organization; 2022. Accessed June 16, 2022. https://www.who.int/publications-detail-redirect/9789240043398

11. World Health Organization WH. *WHO Guideline: Fortification of Maize Flour and Corn Meal with Vitamins and Minerals*. World Health Organization; 2016. Accessed February 11, 2025. https://iris.who.int/handle/10665/251902

12. World Health Organization. *Guideline: Fortification of Food-Grade Salt with Iodine for the Prevention and Control of Iodine Deficiency Disorders*. World Health Organization; 2014. Accessed February 11, 2025. https://www.who.int/publications/i/item/9789241507929

13. World Health Organization. *Guideline: Fortification of Rice with Vitamins and Minerals as a Public Health Strategy*. World Health Organization; 2018. Accessed February 11, 2025. https://www.who.int/publications/i/item/9789241550291

14. Springmann M. Estimates of energy intake, requirements, and imbalances based on anthropometric measurements at global, regional, and national levels and for sociodemographic groups. *in review*.

15. Allen LH, Carriquiry AL, Murphy SP. Perspective: Proposed Harmonized Nutrient Reference Values for Populations. *Advances in Nutrition*. 2020;11(3):469-483. doi:10.1093/advances/nmz096

16. de Jong MH, Melse-Boonstra A, Geleijnse JM, Verkaik-Kloosterman J. Assessment of the prevalence of inadequate iron intakes in premenopausal females based on the reference values of the European Food Safety Authority using cross-sectional food consumption data. *The American Journal of Clinical Nutrition*. 2024;120(1):211-216. doi:10.1016/j.ajcnut.2024.04.033

17. World Bank Group. Population, total. Accessed May 26, 2022. https://data.worldbank.org/indicator/SP.POP.TOTL

18. de Pee S, Tsang BL, Zimmerman S, Montgomery SJ. Chapter 13 - Rice Fortification. In: Mannar MGV, Hurrell RF, eds. *Food Fortification in a Globalized World*. Academic Press; 2018:131-141. doi:10.1016/B978-0-12-802861-2.00013-4

19. World Food Programme. *Handbook for the Production of Extruded Fortified Rice Kernels | World Food Programme*. World Food Programme; 2019. Accessed February 11, 2025. https://www.wfp.org/publications/handbook-production-extruded-fortified-rice-kernels

20. Food and Agriculture Organization of the United Nations (FAO). FAOSTAT: Supply utilization accounts. Published online 2024. Accessed September 19, 2024. https://www.fao.org/faostat/en/#data/SCL

21. United States Agency for International Development (USAID). *Large-Scale Food Fortification Programming Guide*. United States Agency for International Development; 2022. https://agrilinks.org/post/usaid-large-scale-food-fortification-programming-guide-supporting-food-fortification-country

22. Fiedler JL, Afidra R. Vitamin A Fortification in Uganda: Comparing the Feasibility, Coverage, Costs, and Cost-Effectiveness of Fortifying Vegetable Oil and Sugar. 2010;31. Accessed November 7, 2019. https://doi.org/10.1177/156482651003100202

23. Fiedler JL, Macdonald B. A strategic approach to the unfinished fortification agenda: feasibility, costs, and cost-effectiveness analysis of fortification programs in 48 countries. *Food Nutr Bull*. 2009;30(4):283-316. doi:10.1177/156482650903000401

24. World Bank Group. GDP per person employed (constant 2021 PPP $). Published online 2023. Accessed January 15, 2025. https://data.worldbank.org/indicator/SL.GDP.PCAP.EM.KD,

25. United Nations, Department of Economic and Social Affairs. Labor share of GDP (%). Accessed February 11, 2025. https://unstats.un.org/sdgs/dataportal/database

26. Fiedler JL, Puett C. Micronutrient Program Costs: Sources of Variations and Noncomparabilities. *Food Nutr Bull*. 2015;36(1):43-56. doi:10.1177/156482651503600105

27. Adams KP, Gashu D, Zegeye EA, et al. The cost of expanding Ethiopia's salt iodization program to include multiple micronutrients. *Current Developments in Nutrition*. 2025;0(0). doi:10.1016/j.cdnut.2025.107508

28. Adams KP, Jarvis M, Vosti SA, et al. Estimating the cost and cost-effectiveness of adding zinc to, and improving the performance of, Burkina Faso's mandatory wheat flour fortification programme. *Maternal & Child Nutrition*. 2023;19(3):e13515. doi:10.1111/mcn.13515

29. Vosti SA, Jarvis M, Anjorin OM, et al. The costs and the potential allocation of costs of bouillon fortification: The cases of Nigeria, Senegal, and Burkina Faso. *Annals of the New York Academy of Sciences*. 2024;1541(1):181-201. doi:10.1111/nyas.15234
