# Supplementary material for: Impact of large-scale food fortification programmes on micronutrient inadequacies and their implementation costs: a modelling analysis
Source: Lancet Glob Health. 2026 Mar 25;14(5):e762–71. doi: 10.1016/S2214-109X(26)00023-9 (PMC13106045; doi:10.1016/S2214-109X(26)00023-9)
Supplement: Equitable Partnership Declaration [file mmc2.pdf]

THE LANCET
Global Health

Supplementary appendix 2

This Equitable Partnership Declaration (EPD) was submitted by the authors, and we reproduce it as supplied. It has not been peer reviewed. *The Lancet*'s editorial processes have not been applied to the EPD.

Supplement to: Friesen VM, Free CM, Adams KP, et al. Impact of large-scale food fortification programmes on micronutrient inadequacies and their implementation costs: a modelling analysis. *Lancet Glob Health* 2026; published online March 25.
https://doi.org/10.1016/S2214-109X(26)00023-9.

Equitable Partnership Declaration

If any questions do not apply to your study, please indicate "N/A" for "not applicable.
For more information on how to complete this form see the Information for Authors document.

Researcher considerations

1. Please detail the involvement that researchers who are based in the country or countries of study had during a) study design; b) clinical study processes, such as processing blood samples, prescribing medication, or patient recruitment; c) data interpretation; and d) manuscript preparation, commenting on all aspects. If they were not involved in any of these aspects, please explain why.

   This should include a thorough description of their leadership roles in the study. Are local researchers named in the author list or the acknowledgements, or are they not mentioned at all (and, if not, why)? Please also describe the involvement of early career researchers based in the location of the study. Some of this information might be repeated from the Contributors section in the manuscript. Note: we adhere to ICMJE authorship criteria for naming authors on a paper.

**a) Study design:**<br>N/A
**b) Clinical study processes:**<br>N/A
**c) Data interpretation:**<br>N/A
**d) Manuscript preparation:**<br>N/A

2. How was funding used to remunerate and enhance the skills of researchers in the countries of study? And how was funding used to improve research infrastructure at the study sites?

   Potentially effective investments into long-term skills and opportunities within local institutions could include training or mentorship in analytical techniques and manuscript writing, opportunities to lead all or specific aspects of the study, financial remuneration rather than requiring volunteers, and other professional development and educational opportunities.

   Improvements to research infrastructure could include funding extended trial designs (eg, platform trials), establishment of long-term contracts for research staff, building research facilities, and setting up local control of funding allocation.

**Skills:**<br>N/A
**Research infrastructure:**

N/A

3. How did you safeguard the researchers who implemented the study?

   Please describe how you guaranteed safe working conditions for study staff, including provision of appropriate personal protective equipment, protection from violence, and prevention of overworking.

N/A

Benefits to the communities and regions of study

4. How does the study address the research and policy priorities of its location?

   How were the local priorities determined and then used to inform the research question? Who decided which priorities to take forward? Which elements of the study address those priorities?

This is a global study which provides potential benefits to populations of all countries.

5. How will research products be shared in the community of study?

   For instance, will you be providing written or oral layperson summaries for non-academic information sharing? Will study data be made available to institutions in the region(s) of study? The Lancet Global Health *encourages authors to translate the summary (abstract) into relevant languages after paper editing; do you intend to translate your summary?*

The findings and data will be presented in various formats for use to a diverse set of stakeholders. These may include webinars, slide decks, policy briefs, podcasts, etc. We will also plan to engage with and build capacity for country stakeholders through GAIN's presence in low- and middle-income countries.

6. How were individuals, communities, and environments protected from harm?

a) How did you ensure that sensitive patient data were handled safely and respectfully? Was there any potential for stigma or discrimination against participants arising from any of the procedures or outcomes of the study?

N/A

b) *Might any of the tests be experienced as invasive or culturally insensitive?*

N/A

c) *How did you determine that work was sensitive to traditions, restrictions, and considerations of all cultural and religious groups in the study population?*

N/A

d) *Were biowaste and radioactive waste disposed of in accordance with local laws?*

N/A

e) *Were any structures built that would have impacted members of the community or the environment (such as handwashing facilities in a public space)? If so, how did you ensure that you had appropriate community buy-in?*

N/A

f) *How might the study have impacted existing health-care resources (such as staff workloads, use of equipment that is typically employed elsewhere, or reallocation of public funds)?*

N/A

7. Confirm that local ethics review was sought, and please provide the approval number. If not sought, please explain why.

N/A

Secondary analyses

8. Have the data analysed in your study been extracted from another source, such as a national survey, rather than being directly collected by the authors of this paper?

The data anlayzed were provided by globally harmonized datasets of the Global Fortification Data Exchange, the Global Dietary Database, and FAO Food Balance Sheets.

If the authors of this paper were not involved in data collection, how were the findings interpreted with sufficient contextual knowledge?
The Lancet Global Health *believe contextual understanding is crucial for informed data analysis and interpretation.*

The authors have been working with country stakeholders in low- and middle-income countries to understand the data in these settings and their implications. We will continue to engage as specified in #5.

---

9. Please provide the title (eg, Dr/Prof, Mr/Mrs/Ms/Mx), name, and email address of an author who can be contacted about this statement.

Name: Ty Beal
Email: tbeal@gainhealth.org

10. Finally, please provide the title and name of an author from one country of study who has seen and approved this form.

Name: N/A
